# Low level of expression of known deafness genes *Kcne1, Kcnj10* or *Col4a3* is sufficient to maintain hearing in mice

**DOI:** 10.1016/j.heares.2025.109356

**Published:** 2025-07-04

**Authors:** Darcey A Kirwin, Nina Treder, Elisa Martelletti, Daniel R Pentland, Rechal Kumar, Neil J Ingham, Karen P Steel

**Affiliations:** Wolfson Sensory, Pain and Regeneration Centre, King’s College London, London SE1 1UL, UK

**Keywords:** Mouse genetics, Hearing loss, Stria vascularis

## Abstract

*Kcne1, Kcnj10* and *Col4a3* are all expressed in the stria vascularis where they serve critical roles in generating the endocochlear potential. Mutations in any of these three genes are linked to human deafness syndromes for which there are currently no treatments. Here, the hearing ability of three mouse lines carrying mutant alleles in these genes (*Kcne1*^*tm1a*^, *Kcnj10*^*tm1a*^ and *Col4a3*^*tm1a*^) was investigated to assess whether they would develop an auditory phenotype similar to that of human patients. Surprisingly, all three mutant mice had normal hearing, at least up to 6 months of age, assessed by auditory brainstem response thresholds and waveforms and endocochlear potentials. Marginal cell arrangement in the stria vascularis was normal in *Col4a3*^*tm1a/tm1a*^ mice at 8 weeks of age but showed evidence of early cellular disorganisation in *Kcnj10*^*tm1a/tm1a*^ and *Kcne1*^*tm1a/tm1a*^ mice. We found that the *Kcne1*^*tm1a*^, *Kcnj10*^*tm1a*^ and *Col4a3*^*tm1a*^ mutations led to incomplete knockdown of transcript, to 25 % of normal values in each case, indicating these are hypomorphic alleles. These results suggest that partial restoration of *KCNE1, KCNJ10* and *COL4A3* expression in patients affected by mutations in these genes may be sufficient to preserve auditory function.

## 1 Introduction

Hearing impairment is common in the population and can begin at any age. It is a heterogenous disorder where environmental factors contribute, but genetics plays a major role. A total of 156 genes have been identified underlying non-syndromic deafness and 40 % of these are involved in progressive hearing loss (Walls et al., 2025). The only remedies currently widely available are hearing aids and cochlear implants, but these do not restore normal function. There is a large unmet clinical need for approaches which slow down or reverse hearing loss. Recent reports have shown that delivery of otoferlin sequences into the mouse and human inner ear after the onset of hearing loss leads to improved hearing (Akil et al., 2019; Lv et al., 2024; Qi et al., 2024). Another report has shown reversal of hearing impairment associated with stria vascularis dysfunction using a genetic approach to turn on the *Spns2* gene in mouse (Martelletti et al., 2023).

The stria vascularis (SV) is a highly specialised and vascularised tissue located in the cochlear lateral wall that functions to maintain cochlear homeostasis. The SV is mainly composed of marginal, intermediate and basal cells (Hibino et al., 2010). These cells express ion channels and transporters which are critical to SV function. We have selected for study three genes expressed in different cell types in the stria vascularis: *Kcne1, Kcnj10* and *Col4a3*.

KCNE1 is a regulatory subunit of the KCNQ1/KCNE1 K^+^ channel, expressed in the apical membranes of marginal cells (Hibino et al., 2010). It allows the transfer of *K*^+^ ions into the endolymph, generating a high potential, the endocochlear potential (EP) (Nin et al., 2008; Wangemann, 2002). In mice with an inactived *Kcne1* gene (*Kcne1*^*tm1Sfh*^) or a spontaneous mutation of *Kcne1* (*Kcne1*^*pkr*^), the endolymphatic compartments of the inner ear collapse early in postnatal development, between birth and 3 days old, leading to profound deafness and vestibular dysfunction (Letts et al., 2000; Nicolas et al., 2001; Vetter et al., 1996; Vidal et al., 2004). Attempts to prevent the collapse of endolymphatic compartments in *Kcne1*^*tm1Sfh*^ homozygotes using gene therapy were only successful (partially) if the viral vector carrying the gene was introduced before postnatal day 2; by postnatal day 3 it was too late to prevent hearing impairment (Wu et al., 2021). Mutations in *KCNE1* are associated with recessively-inherited Jervell and Lange-Nielsen Syndrome (JLNS) type 2 (Faridi et al., 2019; Ohno et al., 2008; Schulze-Bahr et al., 1997; Tyson et al., 1997; Uysal et al., 2017; Vojdani et al., 2019). JLNS type 2 is characterised by profound congenital bilateral deafness and prolonged cardiac QT interval (Friedmann et al., 1966; Jervell and Lange-Nielsen, 1957).

The inwardly rectifying *K*^+^ channel Kir4.1, encoded by *KCNJ10* and expressed in the intermediate cells (Ando and Takeuchi, 1999; Chen and Zhao, 2014), is also critical for hearing because of its association with both Seizures, Sensorineural deafness, Ataxia, Mental retardation and Electrolyte imbalance (SeSAME) syndrome (Scholl et al., 2009) and Epilepsy Ataxia Sensorineural deafness and Tubulopathy (EAST) syndrome (Bockenhauer et al., 2009; Cross et al., 2013; Freudenthal et al., 2011; Marcus et al., 2002; Parrock et al., 2013; Reichold et al., 2010). Both diseases show recessive inheritance. EAST patients experience a range in the age of onset of hearing impairment from 14 years of age onwards (Bockenhauer et al., 2009). The *Kcnj10*^*tm1Lst*^ mutant mouse shows no immunolabelling of the KCNJ10 protein, no response to sounds, collapse of the endolymphatic compartment and early death (Rozengurt et al., 2003). Marcus and colleagues reported reduced *K*^+^ concentration in scala media and reduced endocochlear potential (Marcus et al., 2002).

Blood supply to the SV is critical to its highly metabolic function. The strial capillaries are surrounded by the strial capillary basement membrane (Sakagami et al., 1982). A major component of this basement membrane is the collagen IV protein network. These collagens, particularly COL4A3, are essential to hearing and are associated with human deafness in Alport syndrome (Mochizuki et al., 1994). Alport syndrome is characterised by haematuria and kidney failure as well as progressive hearing loss (Gubler et al., 1981). One study reported that 156 variants in *COL4A3*, 115 in *COL4A4* and 1585 variants in *COL4A5* cause Alport syndrome (Weber et al., 2016). More recently, the total number of variants is predicted to be even higher (Cerkauskaite et al., 2022). Position 1 Gly substitutions are the most common pathogenic variant (Gibson et al., 2021; Wang et al., 2024) because it destabilises the collagen triple helix (Brodsky and Persikov, 2005). The median age of onset for hearing loss in the human population is 20 years of age (Oka et al., 2014) but a broad spectrum of severity of hearing loss exists (Boeckhaus et al., 2020; Moon et al., 2009). Heterozygous carriers of *COL4A3* mutations have an increased risk of renal dysfunction although not for hearing loss (Solanki et al. 2023; Savige 2022), suggesting that the deafness in Alport syndrome is usually recessively inherited. A mouse mutation of *Col4a3* that causes lack of protein production, *Col4a3*^*tm1Dec*^, shows thickening of the basement membranes of kidney glomeruli from around 4 weeks old leading to premature death (Cosgrove et al., 1996). In the cochlea of the mutants, basement membranes around strial capillaries were thickened but auditory brainstem response (ABR) thresholds were minimally affected (Cosgrove et al., 1998). The lack of COL4A3 protein is linked with abnormal expression of other components of basement membranes including other collagens (Cosgrove et al., 1996; Dufek, Meehan, Delimont, Samuelson, et al., 2020, 2020; Gratton et al., 2005; Meehan et al., 2016). A recent report suggests these strial capillary basement membrane changes are associated with a small drop in endocochlear potential (Smyth et al., 2024).

Together, these three genes represent critical factors for the maintenance of normal hearing, but there are currently no treatments available for the deafness associated with them. We aim to address whether the auditory phenotype of these three mutant mice is similar to that seen in both humans and other mice with mutations in these genes. However, for all three mutant mice we observe normal responses to all assessments of hearing ability at least up to 6 months of age, despite a low level of transcript from the mutant alleles. Structurally, the SV is normal in *Col4a3*^*tm1a/tm1a*^ mice, but *Kcnj10*^*tm1a/tm1a*^ and *Kcne1*^*tm1a/tm1a*^ mice show early signs of cellular disorganisation.

## 2 Methods

### 2.1 Ethics statement

All mouse work was carried out in accordance with UK Home Office regulations and the UK Animals (Scientific Procedures) Act of 1986 under UK Home Office licences, and the study was approved by the King’s College London Animal Welfare and Ethical Review Body. Mice were culled using methods approved under this licence. The mice were maintained in individually ventilated cages at a standard temperature and humidity and in specific pathogen-free conditions. Where possible, all experimental mice were age and sex-matched to controls. Both males and females were studied.

### 2.2 Mice

**Fig. 1:**
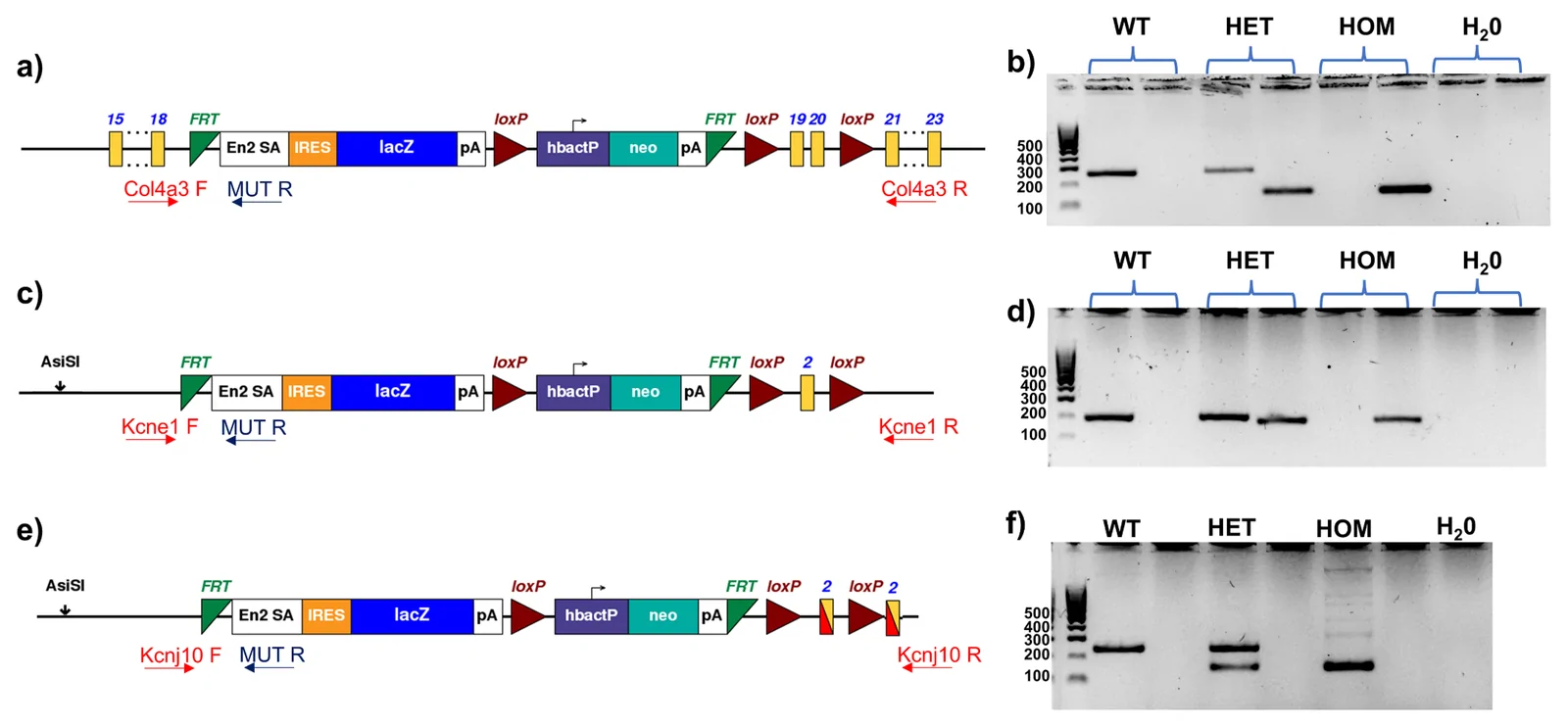
The tm1a knockout-first allele design and genotyping. **(a-b)** *Col4a3*^*tm1a*^, **(c-d)** *Kcne1*^*tm1a*^ and **(e-f)** *Kcnj10*^*tm1a*^ mice. A large, inserted cassette just upstream of a critical exon (yellow box) is intended to interfere with transcription leading to knockdown of gene expression. Allele images **(a, c, e)** are from mousephenotype. org. Green flags represent FRT recognition sites for Flp recombinase activity. Red triangles represent LoxP recognition sites for Cre recombinase activity. The LacZ element and neomycin represent exogenous bacterial DNA (Skarnes et al. 2011). (**b, d, f**) show representative gel photos to illustrate the genotyping results from short-range PCR using the primers indicated by arrows below each schematic; in the mutant allele, gene-specific primers are located too far apart to generate a PCR product. Samples in panel **f** were multiplexed so there are blank lanes between WT, HET, HOM and H_2_0 samples.

*Kcnj10*^*tm1a(KOMP)Wtsi*^ (*Kcnj10*^*tm1a*^; EMMA ID EM:09266), *Kcne1*^*tm1a (EUCOMM)Hmgu*^ (*Kcne1*^*tm1a*^; EMMA ID EM:10850) and *Col4a3*^*tm1a(EUCOMM) Wtsi*^ (*Col4a3*^*tm1a*^; EMMA ID EM:07131) mice carry a large cassette of exogenous DNA inserted in an intron intended to interfere with transcription leading to knockdown of the gene (Skarnes et al., 2011). The disruption cassette for all three alleles is flanked by FRT sites to enable its removal and conversion to the conditional *tm1c* allele. loxP sites flanking the critical exon enable exon removal on exposure to Cre recombinase, creating the *tm1b* allele. A schematic of the designs of these three alleles is shown (Fig. 1). *Kcnj10*^*tm1a*^, *Kcne1*^*tm1a*^ and *Col4a3*^*tm1a*^ mutations were generated and maintained on a C57BL/6N genetic background. For analysis, we crossed the mutations to a targeted repaired allele of *Cdh23*^*ahl*^, *Cdh23*^*ahl*+*em3H*^ (EMMA ID EM:10,890; Mianné et al. 2016) because the *Cdh23*^*ahl*^ variant is known to cause progressive high-frequency hearing loss in C57BL/6N mice (Noben-Trauth et al., 2003; Yasuda et al., 2020). However, the *Col4a3*^*tm1a*^ mutation was also analysed on the original *Cdh23*^*ahl*^ genetic background. Both wildtype (WT) (*Kcnj10*^+*/*+^, *Kcne1*^+*/*+^and *Col4a3*^+*/*+^) and heterozygous (*Kcnj10*^+*/tm1a*^, *Kcne1*^+*/tm1a*^ and *Col4a3*^+*/tm1a*^) mice were used as controls for experimental mice (*Kcnj10*^*tm1a/tm1a*^, *Kcne1*^*t-m1a/tm1a*^ and *Col4a3*^*tm1a/tm1a*^). All three mutants were viable, with 55 homozygous *Kcnj10*^*tm1a/tm1a*^ mice weaned out of a total of 266 offspring, 55 *Kcne1*^*tm1a/tm1a*^ out of 295 offspring, and 25 *Col4a3*^*tm1a/tm1a*^ out of 113 offspring from heterozygous intercrosses. All mutant mice had normal weights, *Kcnj10*^*tm1a/tm1a*^ (14.7 ± 2.4 g), *Kcne1*^*tm1a/tm1a*^ (14.9 ± 1.2 g) and *Col4a3*^*tm1a/tm1a*^ (17.9 ± 1.7 g) at 4 weeks of age compared to their wildtype littermate controls (16.2 ± 3.2 g, 14.7 ± 1.9 g and 16.0 ± 1.3 g) respectively (One-way ANOVA, *p* > 0.05 in all cases).

### 2.3 Genotyping

DNA was extracted from pinna skin by lysing overnight in a solution containing proteinase K. This was used as the template for short-range PCR using primers listed in Table 1 (binding locations indicated in Fig. 1). The WT product was not amplified from the *tm1a* mutant allele because the primers bound to sites which were too far apart for short-range PCR to be successful. The presence of the disruption cassette was checked using a second confirmational reaction where primers were designed to bind to the neomycin gene within the *tm1a* allele. This reaction would only be successful in the presence of the *tm1a* allele, but it cannot distinguish between mice which are heterozygous or homozygote for the *tm1a* allele. The presence of the WT band in combination with the mutant band, as well as a positive neo reaction, confirmed a heterozygote animal. Animals with no WT band but a mutant band, as well as a positive neoreaction, confirmed a homozygote animal.

### 2.4 Auditory-Evoked brainstem response (ABR) recordings

Mice were anaesthetised using intraperitoneal injection of 100 mg/ kg Ketamine (Ketamidor, Chanelle Pharma) and 10 mg/kg Xylazine (Rompun, Bayer Animal Health). Mice were placed into a sound attenuated chamber fitted with a blanket heated to body temperature. Subcutaneous electrodes (NeuroDart; Unimed Electrode Supplied Ltd.) were placed behind each pinna and at the vertex. Evoked responses were recorded to free-field calibrated broadband click stimuli (10 μs duration) and to tonebursts (5 ms duration, 1 ms onset/offset ramp, fixed phase onset) at frequencies of 3, 6, 12, 18, 24, 30, 36 and 42 kHz. The stimuli were presented at 5 dB incremental steps from 0 – 95 dB SPL at a rate of 42.6 stimuli per second. The evoked responses were digitised, amplified and bandpass filtered between 300 and 3000 Hz. 256 responses were averaged for each frequency and intensity combination to produce one waveform. Threshold calls for a particular frequency were defined as the lowest stimulus level to evoke a visually detected waveform. Mice were tested recurrently for ABRs at 4, 8, 14 weeks and 6 months of age. For recurrent procedures, 0.1 mg/ml Atipamezole (Antisedan, Vetoquinol and Orion Pharma) was administered to mice at the end of the ABR to aid with recovery.

### 2.5 Endocochlear potential (EP) recordings

8 week old mice were anaesthetised using intraperitoneal injection of urethane (0.1 mL/10 g body weight of a 20 % w/v solution of urethane). Mice were tested for the absence of pedal withdrawal reflex before surgery. Mice were tracheotomised and fixed into a head holder on a blanket heated to body temperature. The left pinna was removed, and a reference electrode (Ag-AgCl pellet) was placed under the skin of the neck. Surrounding soft tissue and muscle was removed to uncover the left auditory bullae so that a hole could be made, exposing the basal turn of the cochlea. A scalpel was used to make a small incision in the bone of the basal turn lateral wall, and the tip of a 150-mM KCl-filled glass micropipette was inserted into the scala media. The EP was recorded as a stable positive potential compared with the reference electrode.

### 2.6 Lateral wall surface preparation, confocal image acquisition and analysis

The stria vascularis was analysed in 8 week old mice. Inner ears were fixed in formaldehyde (4 % w/v paraformaldehyde) for 2 h. Following fine dissection of the cochlear lateral wall, samples were stained in phalloidin 488 (1:300, ThermoFisher) for 1 hr at room temperature and mounted in ProLong Gold mounting media with DAPI (P36931, Life Technologies) and stored at 4 ^◦^C. Samples were imaged using a Zeiss Imager 710 confocal microscope interfaced with ZEN 2010 software. The 10X air objective (NA 0.3) was first used to capture images along the whole length of the lateral wall for frequency mapping. Approximate frequency regions were determined according to the mouse tonotopic cochlear map ImageJ plugin “Measure Line” and the frequency-place map of Müller et al. (2005) (Hickman et al., 2018; Muller et al., 2005). Following this, the plan-APOCHROMAT 40X Oil DIC objective (NA 1.3) was used to image frequency regions corresponding to 3, 12, 18, 24 and 42 kHz. Image stacks were taken throughout the SV with a z-step of 0.2 μm. For image analysis, gaps (red arrows, Fig. 7) were defined as a discrete disruption to the ordered hexagonal arrangement of marginal cells that can contain one or more nuclei but did not run along the border of the SV. Anomalies (white arrows) were defined as an unusual arrangement of marginal cells with one or more nuclei that ran along the border of the SV. For quantification of gaps along the whole length of the SV in *Kcnj10*^*tm1a/tm1a*^ and *Kcne1*^*tm1a/tm1a*^ mice and littermate controls, the 20X air DIC objective (NA 0.8) was used to image the whole length of the SV with a z-step of 0.5 μm. Adjacent sections were stitched together using the tile scan setting (online stitching, 10 % overlap between frames). Following image acquisition, the whole length of the stria was divided into 5 % equal divisions and the absolute number of gaps from each 5 % sector were counted and tabulated using the CellCounter plugin in ImageJ.

### 2.7 RNA quantification

4 week old mice were culled and whole inner ears were rapidly dissected in RNALater (AM7024, ThermoFisher) and snap frozen in liquid nitrogen. Total RNA was isolated from one inner ear per mouse and extracted using the Lexogen SplitRNA kit (SKU 008.48, Lexogen). A quality control step was taken using a Nanodrop spectrophotometer to measure RNA purity. RNA was normalised to the same concentration in each sample for cDNA synthesis. For cDNA creation, RNA was first treated with an ezDNase enzyme (11,766,050, Invitrogen) and then retrotranscribed using a first-strand cDNA synthesis reaction mix (Invitrogen). Digital droplet PCR (ddPCR) was used to measure the quantity of *Kcnj10, Kcne1* or *Col4a3* mRNA relative to the housekeeping gene Hypoxanthine-guanine phospharibosyltransferase (*Hprt)* used as a calibrator (probe ID Mm00446968_m1, ThermoFisher). Probes for *Kcnj10* (Mm00445028_m1, ThermoFisher), *Kcne1* (off-catalogue probe designed to cover the protein coding region in exon 2, ThermoFisher) and *Col4a3* (Mm00483669_m1, ThermoFisher) were all selected to bind downstream of the *tm1a* cassette. All equipment and consumables were designed to fit with the Bio-Rad QX100™ Droplet Digital PCR system and was carried out following manufacturer’s instructions.

### 2.8 Protein quantification

Western blot data from *Kcnj10*^*tm1a/tm1a*^ and *Kcne1*^*tm1a/tm1a*^ mice was collected from 8 week old mice. Both left and right inner ears were collected from mutants and wildtype controls. Protein concentration was measured using Pierce™ BCA protein assay according to manufacturer’s instructions (Thermo, ZA381473). Protein lysates (40ug) were resolved by 4–12 % SDS-PAGE for KCNE1 or a 10 %, Mini-PROTEAN TGX precast gel (Bio-Rad, 456–1033) for KCNJ10 and transferred onto PVDF membrane using a Trans-Blot Turbo Transfer system. After transfer the membranes were blocked with skimmed milk powder in TBST and incubated with primary antibodies to KCNE1 (rabbit polyclonal anti-KCNE1; 1:400, Ab180843, Abcam), KCNJ10 (rabbit polyclonal anti-Kir4.1; 1:1000, 12,503–1-AP, Protein-tech) and GAPDH (mouse monoclonal anti-GAPDH; 1:5000, Ab8245, Abcam). The secondary antibodies were then applied: anti-rabbit HRP conjugate (Goat anti-rabbit IgG; AB97051, Abcam) and anti-mouse HRP conjugate (Goat anti-mouse IgG; AB205719, Abcam). For chemiluminescence reaction Amersham ECL Prime Western Blotting Detection kit (18,206,643, Cytiva) was used and imaged with Invitrogen™ iBright™ CL1500 Imaging System. Gapdh was used as a loading control and for normalisation of the Kcne1 and Kcnj10 band intensity quantification. To determine band size, the molecular weight marker used was Precision Plus Protein Dual Colour Standards (Bio-Rad, 1610,374). The Analyze>Gel plugin feature in Fiji was used to quantify the intensity of bands.

### 2.9 Immunohistochemistry in paraffin sections

Inner ears from 4 week old *Col4a3*^+*/*+^ and *Col4a3*^*tm1a/tm1a*^ mice were dissected out and fixed in 4 % paraformaldehyde for 2 hrs. These were washed twice with PBS and decalcified in 0.1 M EDTA (pH 7.4) until soft. Samples were dehydrated in increasing concentrations of EtOH washes prior to embedding in paraffin wax. Paraffin sections were cut to a thickness of 8 µm and mounted on superfrost plus slides (Epredia). Prior to staining, slides were left for 30 min at 60 ^◦^C before dewaxing. Slides were dewaxed in xylene (2 × 5-minute washes) before being washed in 100 % EtOH (4 × 2-minute washes). Sections were then incubated with 3 % H_2_O_2_ for 10 min. An antigen retrieval step was undertaken by heating slides to 90 ^◦^C in citrate buffer (0.1 M, adjusted to pH 2.0) for 6 min. Sections were then incubated in 0.1 M glycine/6 M urea solution, pH 3.2 for 30 min at room temperature. Sections were blocked in a solution made up of 2 g BSA (Sigma A4503), 20 ml 0.5 M tris buffered saline (TBS), 180 ml H_2_0 and 2 ml 10 % sodium Azide for 5 min prior to incubation with the primary antibody (Rat anti-COL4A3, Chondrex at 1:100 dilution in blocking solution) overnight. Sections were then washed three times in 1X tris buffered saline (TBS) and incubated with goat anti-rat biotinylated secondary antibody at 1:200 dilution (Vector Labs, BA-9401–0.5) for 60 min. Following, sections were washed in TBS and incubated in Streptococcus ABR-Horseradish peroxidase (Vector Labs, PK6100 Vectastain Elite ABC kit), prepared according to manufacturer’s instructions, for 30 min at room temperature. Finally, sections were left to develop in 3,3´-diaminobenzidine tetrahydrochloride (DAB) solution (Sigma, D5637) for 10 min. A Haemalum counterstain was applied before imaging. Stained slides were examined, and images were obtained using an AxioCam HRc camera mounted on a Zeiss microscope and objective numerical aperture 1.25.

### 2.10 Electrocardiogram (ECG) recordings

ECGs were recorded in *Kcne1*^*tm1a*^ mutants using the method described in a previous publication on long QT syndrome (Casimiro et al., 2001). Mice were anaesthetised using intraperitoneal injection of 100 mg/kg Ketamine (Ketamidor, Chanelle Pharma) and 10 mg/kg Xylazine (Rompun, Bayer Animal Health) and placed onto a mat heated to body temperature. Three subcutaneous limb leads were positioned to record the EGC in a limb lead II configuration for a duration of 10 min, via a TDT RA4LI / RA4PA low impedance headstage / preamplifier using a custom Matlab (R2023a, The Mathworks Inc) script driving a TDT RZ6 multifunction processor. For analysis, R-Peaks were located using the Matlab *findpeaks* function. A section of response trace, 65.5 ms before each R-peak through to 98.3 ms after the R-peak (long enough to encompass the whole ECG response) was summed and averaged to create the mean trace. The Q-wave was defined as the negative deflection before the R-Peak (defined as the point of inflection where the trace inflects towards R, if no negative deflection is present). The T-wave was defined as the point where the gradient reduced to zero after the T-wave peak.

### 2.11 Urine analysis

Urine was collected from *Col4a3*^*tm1a/tm1a*^ and littermate controls at both 8 weeks and 6 months of age. Urine was also collected from *Kcnj10*^*tm1a/tm1a*^ mice and littermate controls at 6 and 12 weeks of age. Mice were removed from the home cage and placed onto a plastic mat. Urine was collected from the mat and transferred onto a Siemens 8 G Multistix Urianalysis test strip to test for abnormalities in glucose, ketone, specific gravity, blood, pH, protein, nitrite and leukocytes in urine.

### 2.12 Statistics

All datasets were tested for normality using a Shapiro-Wilk test and tested for homogeneity of variance using an F test or Brown-Forsythe test. For datasets with normal distribution and equal variance, comparisons were tested for significance using either an Unpaired *t*-test or a two-way ANOVA with Tukey post-hoc for multiple comparisons. For datasets where *p* < 0.05 (Shapiro-Wilk) and/or *p* < 0.05 (F test or Brown-Forsythe), comparisons were tested for significance using a Mann-Whitney comparison of ranks or a Mixed-effects analysis with Fisher’s LSD test for pairwise comparisons unless otherwise stated. Standard deviation is calculated as the square root of the variance, where variance is the addition of the square of the difference between each value and the sample mean divided by N – 1.

## 3 Results

### 3.1 Mutant mice have normal ABR thresholds and wave 1 amplitude and latency

*Kcne1, Kcnj10* and *Col4a3* are all expressed in the SV and are associated with deafness in both humans and mice carrying mutations in these genes. Mutations in *KCNE1* are associated with profound bilateral congenital deafness. Mutations in either *KCNJ10* or *COL4A3* are associated with progressive high frequency hearing impairment. It is unclear whether gene therapy or other treatments could be used to reverse the hearing impairment associated with mutations in any of these genes. We therefore aimed to characterise the hearing impairment in these alleles (*Kcnj10*^*tm1a*^, *Kcne1*^*tm1a*^ and *Col4a3*^*tm1a*^) to determine a suitable time point to turn gene expression back on to reverse the hearing impairment (as in Martelletti et al. 2023). We expected that all three mutant mouse lines would have raised ABR thresholds compared to littermate controls.

**Fig. 2:**
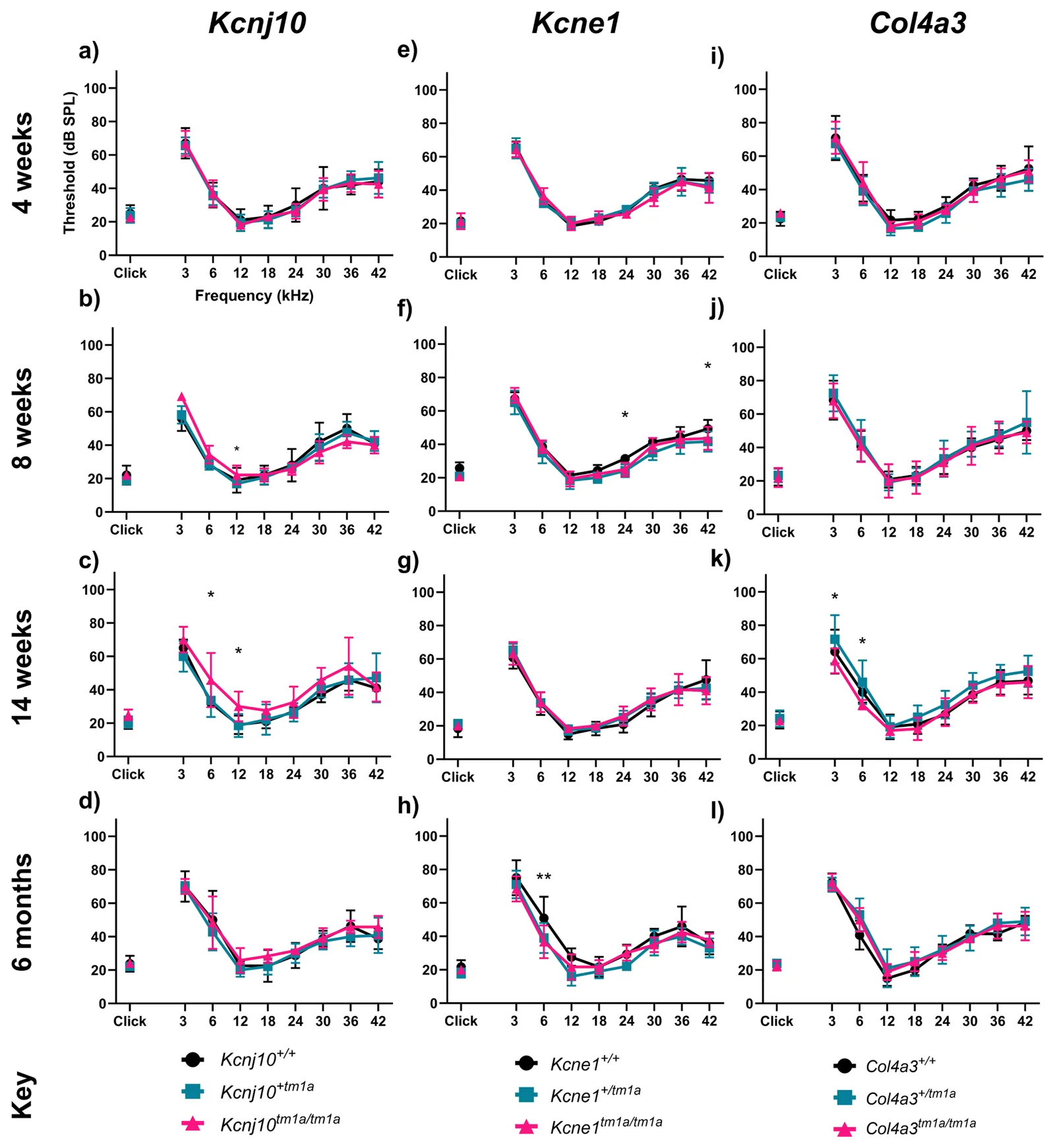
Mutant mice show normal auditory thresholds. Mean ABR thresholds are plotted for homozygous (magenta) *Kcnj10*^*tm1a/tm1a*^ (*n* = 8 at 4 weeks, 7 and 8 weeks and 6 up to 6 months) **(a-d)**, *Kcne1*^*tm1a/tm1a*^ (*n* = 7 up to 6 months) **(e-h)** or *Col4a3*^*tm1a/tm1a*^ (*n* = 5 up to 14 weeks and 4 at 6 months) **(i-l)** mice at 4, 8, 14 weeks and 6 months of age. These mice were tested alongside respective heterozygote (cyan) *Kcnj10*^+*/tm1a*^ (*n* = 8 up to 8 weeks and 7 up to 6 months), *Kcne1*^+*/tm1a*^ (*n* = 6 up to 8 weeks and 5 up to 6 months) and *Col4a3*^+*/tm1a*^ (*n* = 6 up to 14 weeks and 5 at 6 months) as well as respective wildtype littermate (black) controls *Kcnj10*^+*/*+^ (*n* = 5 up to 14 weeks and 4 at 6 months), *Kcne1*^+*/*+^ (*n* = 7 up to 8 weeks and 6 up to 6 months) and *Col4a3*^+*/*+^ (*n* = 6 up to 6 months). These mice were all homozygous for the repaired *Cdh23*^*ahl*+*em3H*^ allele on a C57BL/6N background. Data presented as mean ABR threshold ± SD. Comparisons were tested for significance using a twoway ANOVA with Tukey post-hoc for multiple comparisons. For this figure, where data were not normally distributed a log transformation was applied before twoway ANOVA. **(b)**. * *p* < 0.05, ** *p* < 0.01. The few statistically significant points were inconsistent across age, of small effect size, and show changes in either direction suggesting they were not biologically relevant.

**Fig. 3:**
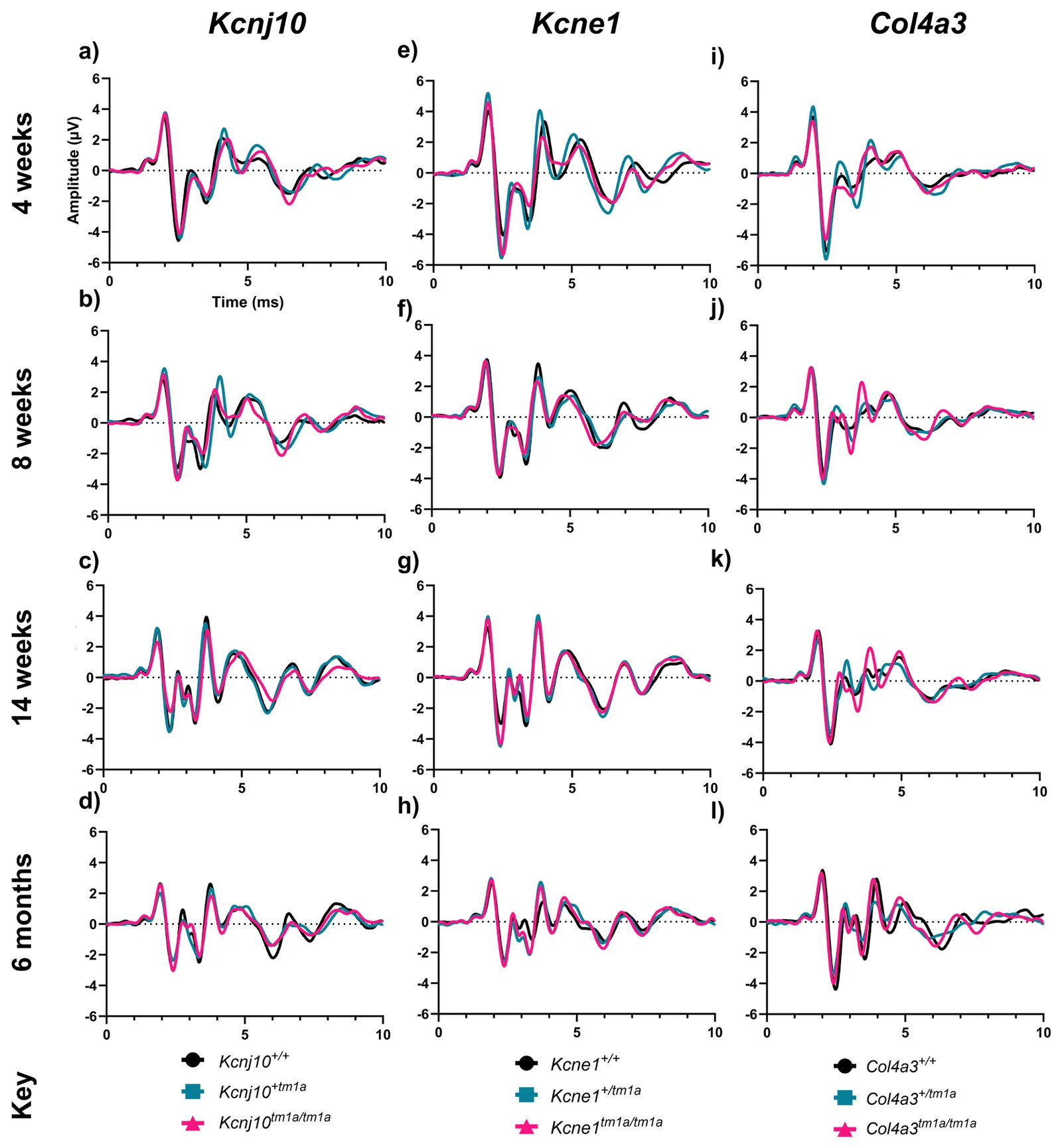
Mutant mice show normal waveform responses. Mean waveform responses to clicks (80 dB SPL) are plotted for homozygous (magenta) *Kcnj10*^*tm1a/tm1a*^ **(a-d)**, *Kcne1*^*tm1a/tm1a*^ **(e-h)**, or *Col4a3*^*tm1a/tm1a*^ **(i-l)** mice aged 14 weeks. Mutant mice were tested alongside heterozygote (cyan) and wildtype (black) littermate controls. Data presented as mean click response waveform. The numbers of mice tested for all genotypes is the same as in Fig. 2.

**Fig. 4:**
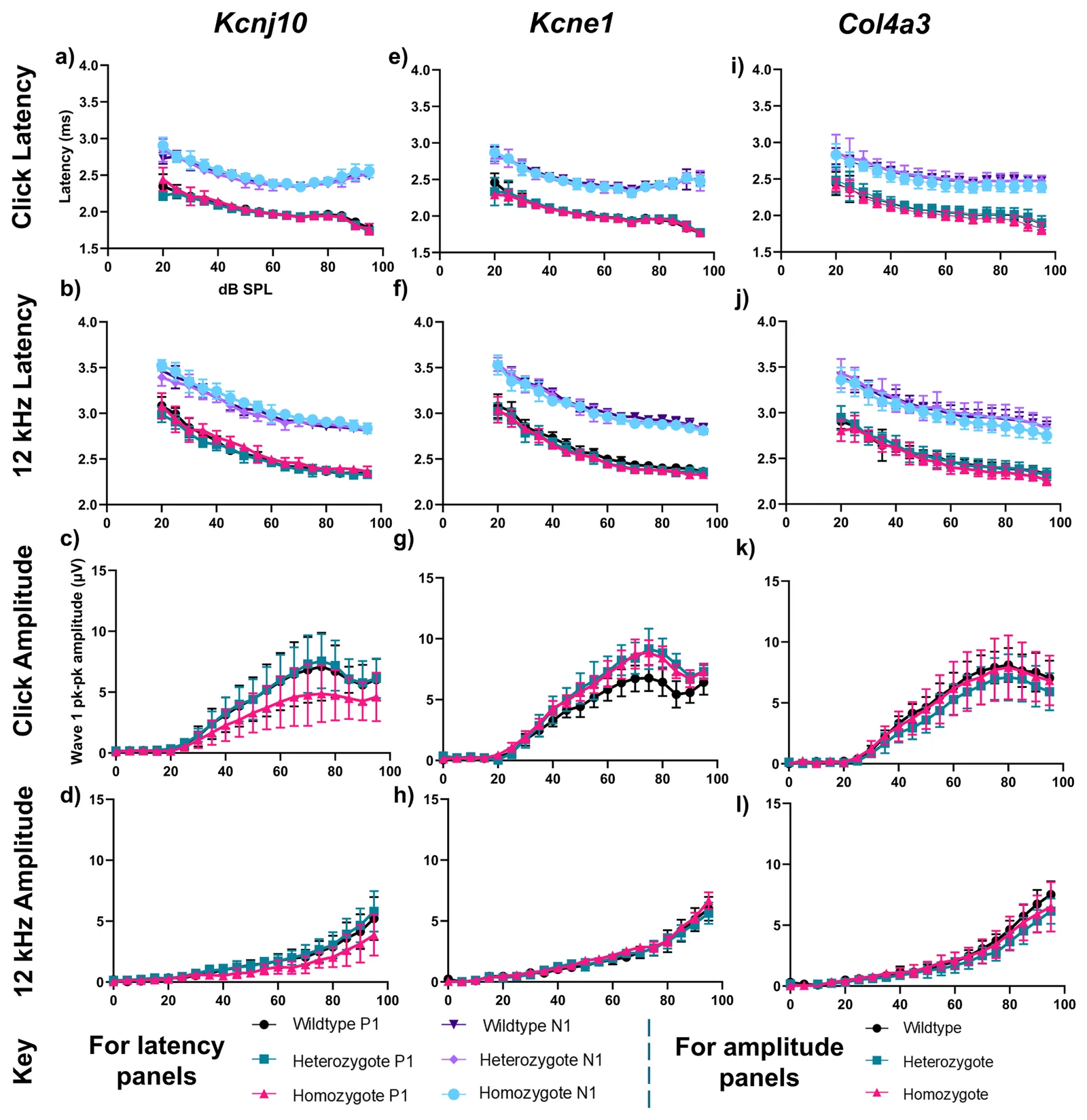
Normal wave 1 amplitude and latency in mutant mice. The average wave 1 latency (P1 and N1) and peak-to-peak amplitude for both click and 12 kHz stimuli are plotted for homozygous *Kcnj10*^*tm1a/tm1a*^ **(a-d)**, *Kcne1*^*tm1a/tm1a*^ **(e-h)** or *Col4a3*^*tm1a/tm1a*^ **(i-l)** mice at 14 weeks of age. Mutant mice were tested alongside heterozygous and wildtype littermate controls. The colour code is given in the key. Data presented as mean ± SD. There were no significant differences between WT and mutant values using a Mann-Whitney or unpaired t-test.

To test this, ABRs were recorded at intervals between 4 weeks and 6 months of age to track any changes in hearing thresholds in individual mice as they aged. Mutant mice were tested alongside littermate controls. Surprisingly, all mutant mice had normal ABR thresholds between 4 weeks and 6 months of age (Fig. 2). The average waveform was plotted for click, 12, 18 and 24 kHz frequency tones at 4, 8, 14 weeks and 6 months of age but no obvious differences were observed between mutants and controls (click data shown in Fig. 3). To quantify changes to wave 1 amplitude or latency, a detailed analysis was performed for clicks and 12 kHz frequency tones at 14 weeks of age, but no significant differences were found between WT and mutant mice at any stimulus level (Fig. 4). During handling, mutant mice did not display any overt signs of abnormal behaviour suggesting normal vestibular function.

**Fig. 5:**
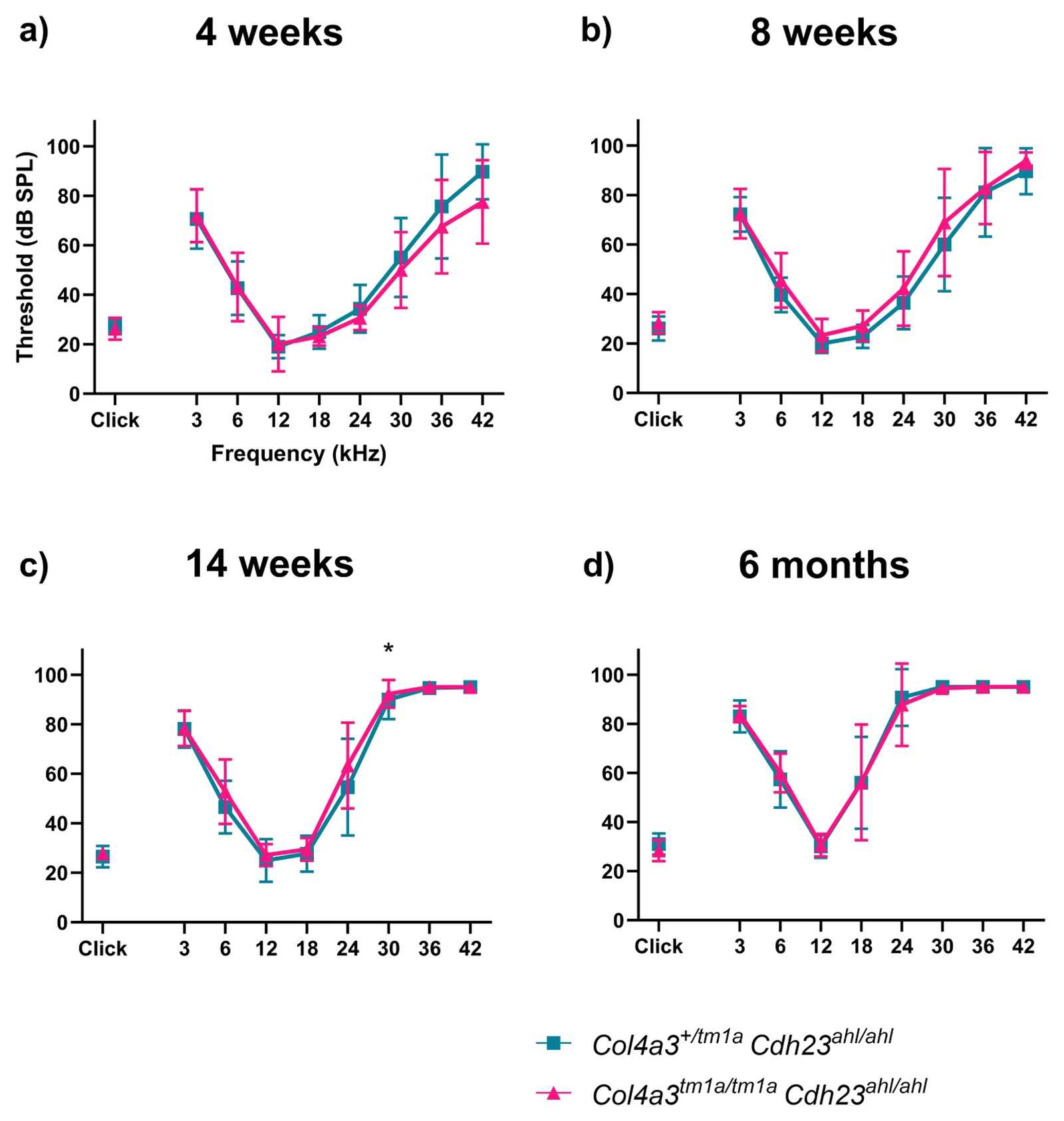
The *Cdh23*^*ahl*^ background does not affect auditory thresholds in *Col4a3*^*tm1a/tm1a*^ mutants. Mean ABR thresholds are plotted for homozygous (magenta) *Col4a3*^*tm1a/tm1a*^*Cdh23*^*ahl/ahl*^ mice (*n* = 9) (**a-d**) at 4, 8, 14 weeks and 6 months of age. Mice were tested alongside heterozygous *Col4a3*^+*/tm1a*^ *Cdh23*^*ahl/ahl*^ (cyan) littermate controls (*n* = 15). Data presented as mean ABR threshold ± SD. Each individual age group was tested for normality using a Shapiro-Wilk test. Comparisons were tested for significance using a two-way ANOVA with Tukey post-hoc for multiple comparisons, * *p* < 0.05. Where data were not normally distributed a log transformation was applied before two-way ANOVA **(d)**. The statistically significant point was inconsistent across age and of small effect size, suggesting it was not biologically relevant.

The main ABR recordings were carried out on a C57BL/6N genetic background that carried the repaired allele of *Cdh23, Cdh23*^*ahl*+*em3H*^, because the usual allele at this locus, *Cdh23*^*ahl*^, leads to slowly progressive hearing loss with age. Using the repaired *Cdh23* allele has led to a loss of phenotype in other situations (Newton et al., 2023), so we tested the *Col4a3*^*tm1a*^ line on the original *Cdh23*^*ahl*^ genetic background in addition. However, no significant differences in ABR thresholds were found between *Col4a3*^*tm1a/tm1a*^ and *Col4a3*^+*/tm1a*^ on the unrepaired *Cdh23* background (Fig. 5a-d).

### 3.2 Mutant mice have a normal endocochlear potential

**Fig. 6:**
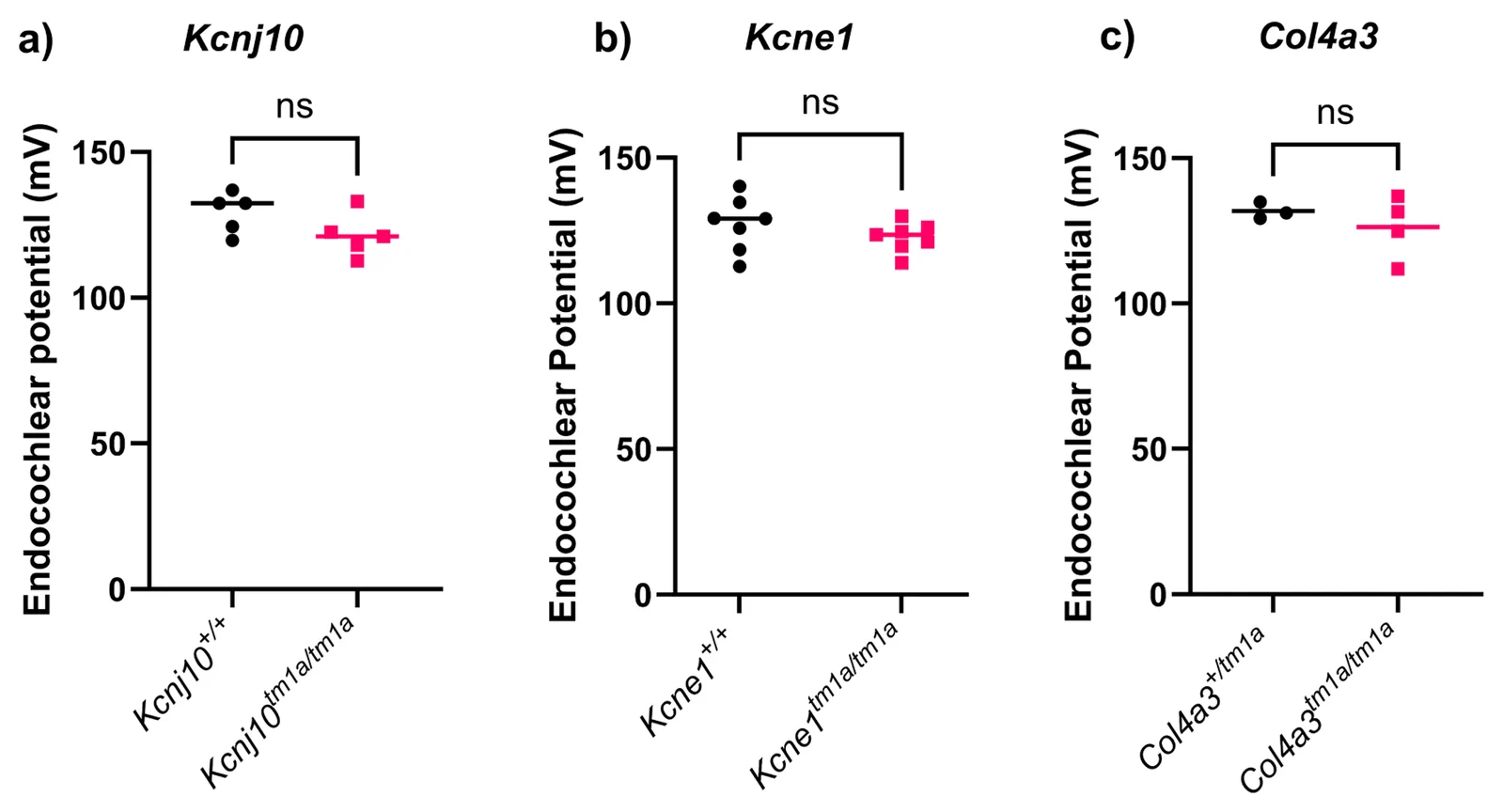
Mutant mice have a normal EP at 8 weeks of age. **(a)** *Kcnj10*^*tm1a/tm1a*^ (*n* = 5), **(b)**, *Kcne1*^*tm1a/tm1a*^ (*n* = 7) and **(c)** *Col4a3*^*tm1a/tm1a*^ (*n* = 4) have normal EP magnitudes of between 100 – 140 mV and there were no significant differences between mutants and controls using an unpaired *t*-test. *p* > 0.05 for all comparisons. Data presented as individual datapoints with mean bar overlaid. Littermate controls were wildtypes (*Kcnj10*^+*/*+^*n* = 5, *Kcne1*^+*/*+^*n* = 7) **(a**,**b)** or heterozygotes (*Col4a3*^+*/tm1a*^*n* = 3) **(c)**.

The SV is responsible for generating the endocochlear potential (EP) (Tasaki and Spyropoulos, 1959). This provides an electrochemical driving force for ions to enter the hair cell when mechanoelectrical transduction channels are opened in stereocilia bundles by sound (von Békésy, 1952) Therefore, recording EP serves as a measure for SV function *in vivo*. We predicted that SV function might be impaired in these three mutant mice because studies from mice with different alleles have reported reduced EP (Marcus et al., 2002; Smyth et al., 2024) or other signs of strial dysfunction such as collapse of scala media (Wangemann et al., 2004; Wu et al., 2021). EP was measured in *Kcnj10*^*tm1a/tm1a*^, *Kcne1*^*tm1a/tm1a*^ and *Col4a3*^*tm1a/tm1a*^ and littermate controls at 8 weeks of age. All mice showed normal EP values of between 110 and 140 mV (Fig. 6) suggesting intact lateral wall function.

### 3.3 Abnormal structure and arrangement of marginal cells in Kcnj10^tm1a/tm1a^ and Kcne1^tm1a/tm1a^ mice

**Fig. 7:**
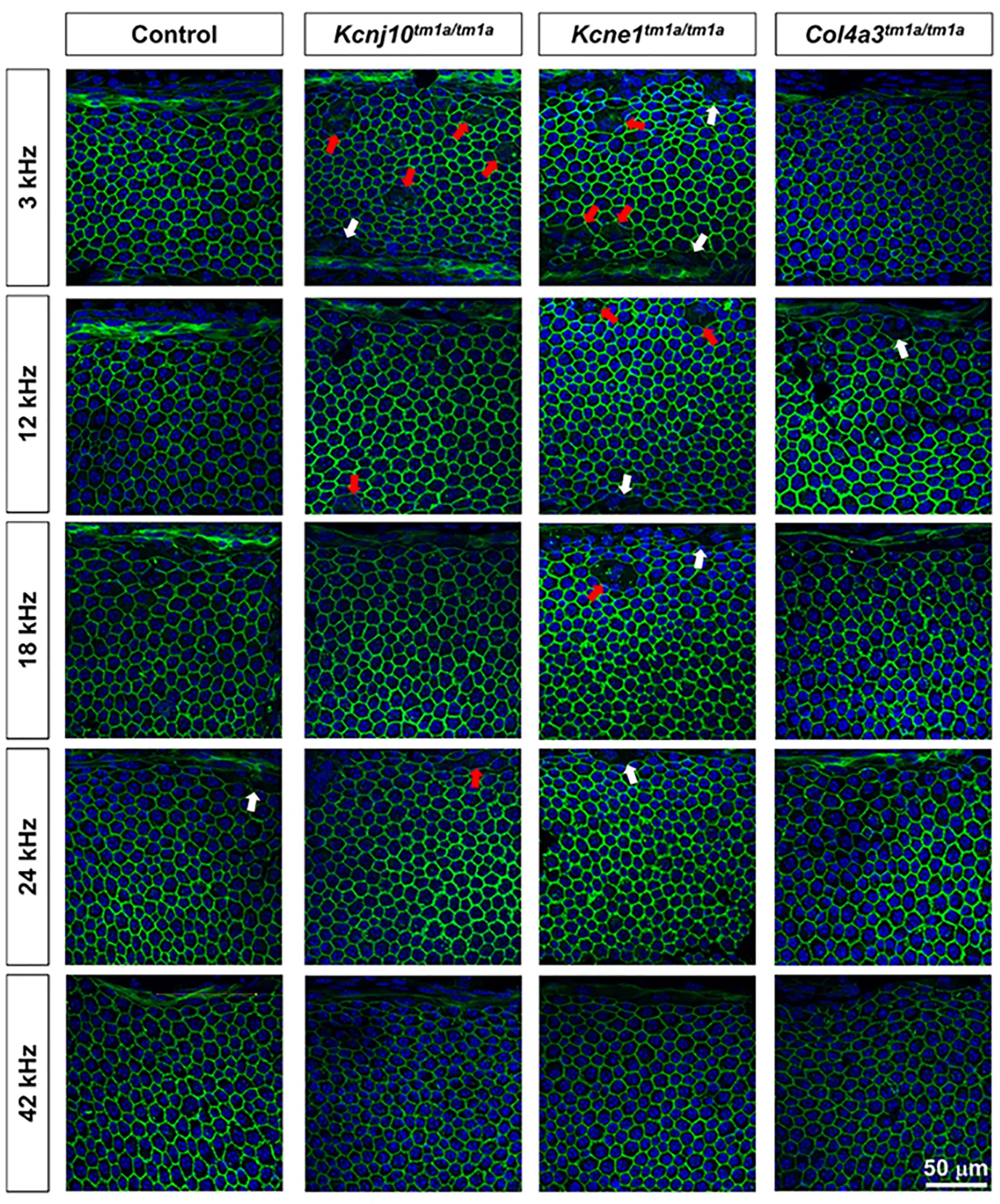
Abnormal arrangement of marginal cells in the stria vascularis of mutant mice. Whole mount preparations of the lateral wall of *Kcnj10*^*tm1a/tm1a*^, *Kcne1*^*tm1a/tm1a*^ and *Col4a3*^*tm1a/tm1a*^ mice compared with a representative control mouse (*Col4a3*^+*/tm1a*^) aged 8 weeks. Samples were stained with phalloidin (green) and DAPI (blue). Red arrows indicate gaps in phalloidin labelling pattern and white arrows indicate anomalies in the arrangement of cells at the edge of the stria vascularis.

As all three genes in this study are expressed in the SV, whole mount SV preparations from mutant and control mice aged 8 weeks were examined after staining with phalloidin (green) to label cell boundaries and DAPI (blue) to label nuclei. Discrete gaps (red arrows) were observed in the regular arrangement of phalloidin labelling in *Kcnj10*^*tm1a/tm1a*^ and *Kcne1*^*tm1a/tm1a*^ mice, but not in *Col4a*^*tm1a/tm1a*^ mice (Fig. 7). Gaps often contained multiple nuclei rather than appearing as a single enlarged cell, but it is currently unclear whether these regions are holes or filled with cellular components. This may be a sign of early degeneration of the SV or patchy loss of F-actin from the marginal cell tight-junctions, but it was not sufficient to cause hearing impairment in these mice. Anomalies, defined in this report as an irregular arrangement of the marginal cells at the border of the SV (white arrows), were also observed, most often in extremely apical (3 kHz) regions, in all mutant mouse lines as well as in heterozygote and WT littermate controls, suggesting that this is a normal feature of the SV in this frequency region. A small number of anomalies were also observed in more basal regions of all mutant mouse lines.

**Fig. 8:**
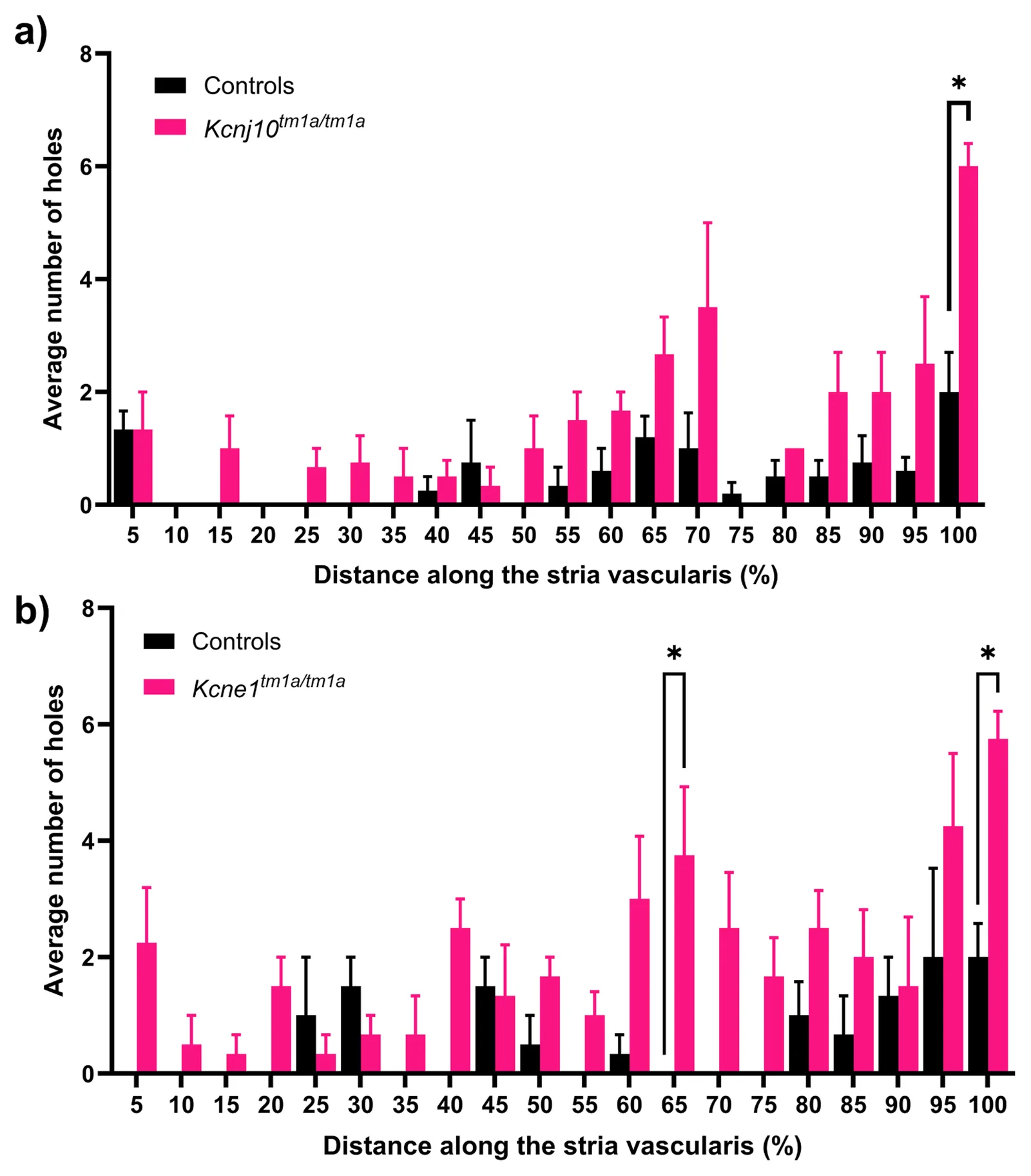
*Kcnj10*^*tm1a/tm1a*^ and *Kcne1*^*tm1a/tm1a*^ mice have sparse gaps in phalloidin labelling which are distributed throughout the entire length of the stria vascularis. The average number of gaps along the length of the stria vascularis in **(a)** *Kcnj10*^*tm1a/tm1a*^ mice (*n* = 4), **(b)** *Kcne1*^*tm1a/tm1a*^ mice (*n* = 4), **(a)** *Kcnj10*^+*/*+^and *Kcnj10*^+*/tm1a*^ control mice (*n* = 5) and **(b)** *Kcne1*^+*/tm1a*^ control mice (*n* = 3). In any circumstance where a 5 % region was damaged during tissue processing, it was omitted from the analysis. Data presented as mean ± SD. For this figure, where data were not normally distributed a log transformation was applied before two-way ANOVA. Sidak’s multiple comparisons were used for post-hoc analysis * *p* < 0.05.

To investigate the distribution of gaps in more detail, the absolute number of gaps throughout the SV was quantified. Both *Kcnj10*^*tm1a/tm1a*^ (Fig. 8a) and *Kcne1*^*tm1a/tm1a*^ mice (Fig. 8b) have gaps distributed throughout the entire length of the SV. This may represent early signs of cellular degeneration. The location of these appears to be scattered along the cochlear duct and not clustered to a particular frequency region. However, for both mutant mice there is a tendency for there to be the highest number of gaps in the most apical 95–100 % of the distance from the base of the cochlea; in this region the average number of holes in the SV of both *Kcnj10*^*tm1a/tm1a*^ and *Kcne1*^*tm1a/tm1a*^ mice was significantly higher than littermate controls. Interestingly, when the entire length of the SV of both wildtype and heterozygote littermate controls was analysed, a very small number of gaps was also observed distributed along the length of the SV (Fig. 8a,b, black bars). This suggests that a small amount of abnormal arrangement of marginal cells in the SV is a normal feature of the mouse cochlea and does not lead to hearing impairment.

### 3.4 Incomplete knockdown of transcript in the inner ear could explain normal hearing

**Fig. 9:**
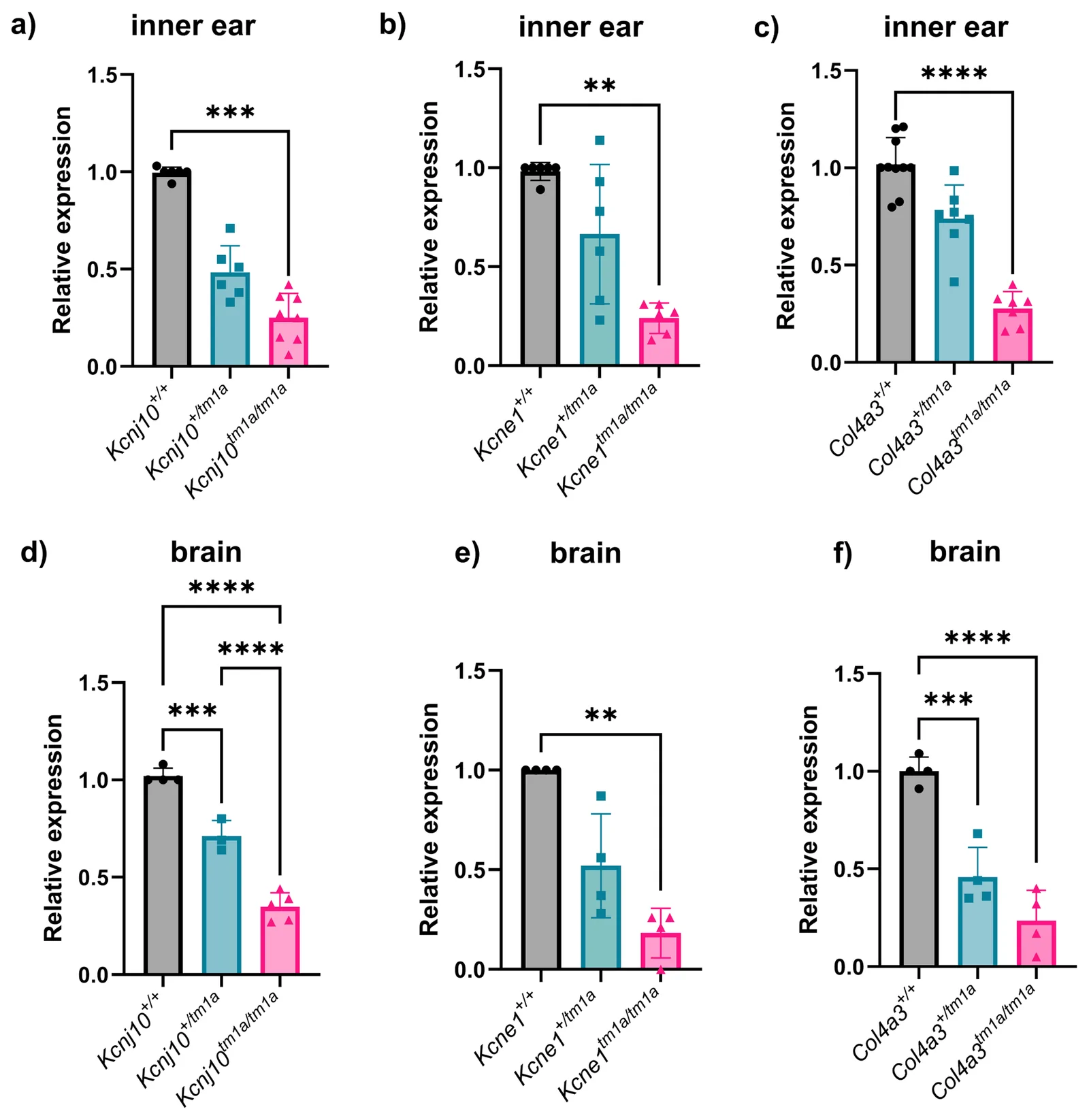
Gene expression in the inner ear and brain. Relative inner ear mRNA expression level at P28 of **(a)** *Kcnj10* in *Kcnj10*^+*/*+^(*n* = 6), *Kcnj10*^+*/tm1a*^ (*n* = 7) and *Kcnj10*^*tm1a/tm1a*^ (*n* = 8); **(b)**, *Kcne1* in *Kcne1*^+*/*+^ (*n* = 6), *Kcne1*^+*/tm1a*^ (*n* = 6) and *Kcne1*^*tm1a/tm1a*^ (*n* = 6); and **(c)** *Col4a3* in *Col4a3*^+*/*+^(*n* = 10), *Col4a3*^+*/tm1a*^ (*n* = 7) and *Col4a3*^*tm1a/tm1a*^ (*n* = 7). Expression was also quantified in brain tissue for **(d)** *Kcnj10* in *Kcnj10*^+*/*+^(*n* = 4), *Kcnj10*^+*/tm1a*^ (*n* = 3) and *Kcnj10*^*tm1a/tm1a*^ (*n* = 5); **(e)** *Kcne1* in *Kcne1*^+*/*+^ (*n* = 4), *Kcne1*^+*/tm1a*^ (*n* = 4) and *Kcne1*^*tm1a/tm1a*^ (*n* = 4); and **(f)** *Col4a3* in *Col4a3*^+*/*+^(*n* = 4), *Col4a3*^+*/tm1a*^ (*n* = 4) and *Col4a3*^*tm1a/tm1a*^ (*n* = 4). Comparisons were tested for significance using the Kruskall-Wallis test (a, b, c, e) or a One-Way ANOVA (d, f), ** *p* < 0.01, *** *p* < 0.001, **** *p* < 0.0001.

Normal hearing and relatively normal appearance of the SV in all mutant mice was unexpected. This prompted us to investigate the level of knockdown of mRNA expression in mutant inner ears and brain tissue (Fig. 9). The *tm1a* allele contains a large cassette of DNA just upstream of a critical exon, which is intended to disrupt transcription (Skarnes et al. 2011). ddPCR was used to estimate the level of mRNA expression in each genotype of the three mutant lines. *Kcnj10* mRNA expression was significantly reduced to 25.1 % in the inner ear and 34.1 % in brain tissue of homozygotes compared to WT controls (Fig. 9a, d). *Kcne1* mRNA expression was significantly reduced to 24.4 % in the inner ear and 18.3 % in brain tissue of *Kcne1*^*tm1a/tm1a*^ compared to WT values (Fig. 9b, e). *Col4a3* inner ear mRNA was significantly reduced to 25.9 % in the inner ear and 23.5 % in brain tissue of *Col4a3*^*tm1a/tm1a*^ compared to WT values (Fig. 9c, f). The total expression of *Kcne1* and *Col4a3* in the brain was low for all genotypes which is similar to other reports of these genes in mouse brain (Goldman et al., 2009; Moreno et al., 2022). For all datasets, mice which are heterozygous for the *tm1a* allele had an intermediate level of knockdown (Fig. 9a-f). Despite this reduction, no hearing impairment was observed suggesting that roughly 25 % of normal gene expression of these three genes in the inner ear is sufficient to preserve auditory function.

### 3.5 Level of knockdown of protein expression in the inner ear varies for each mutant

**Fig. 10:**
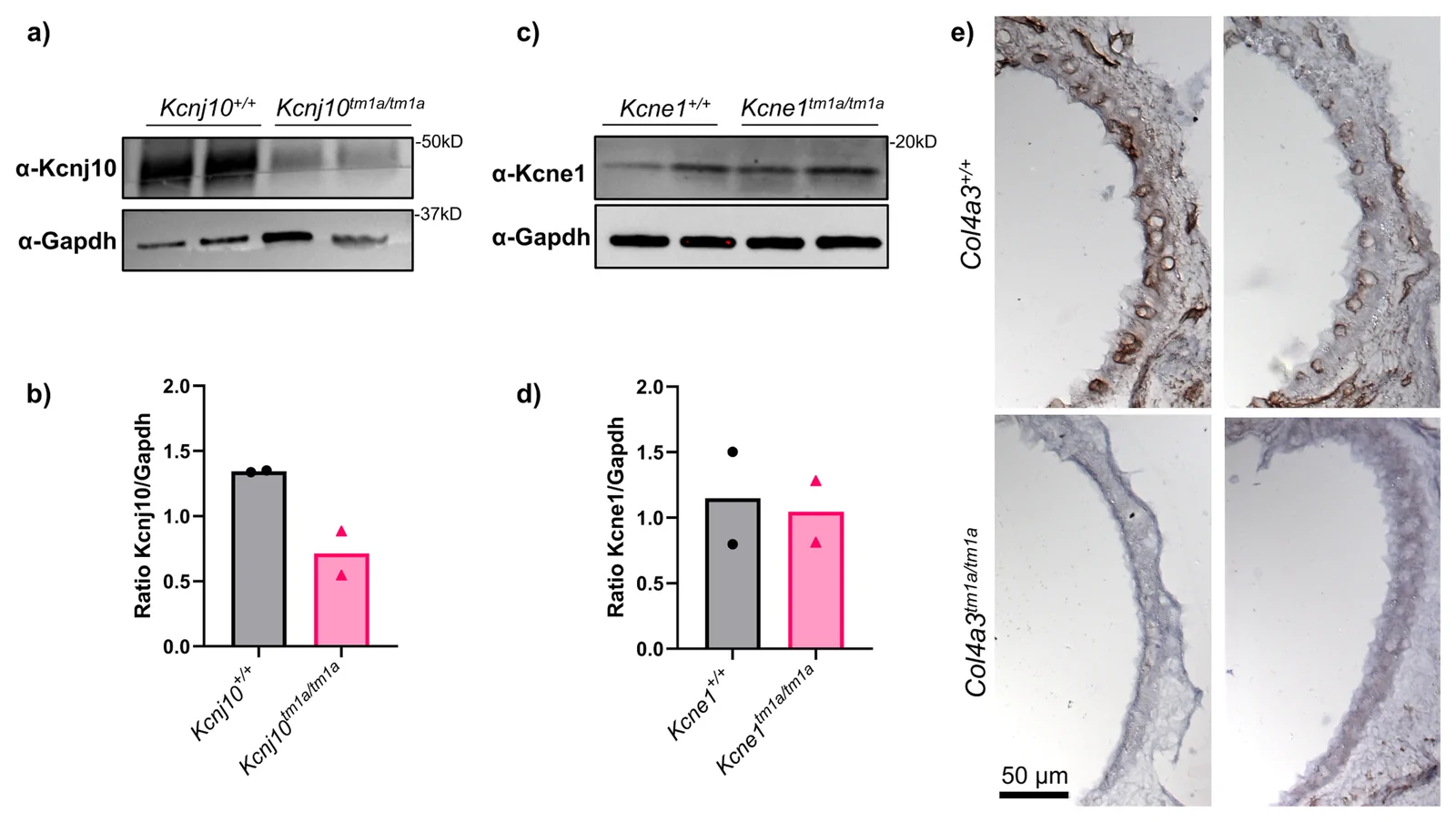
Protein expression in the inner ear. Inner ear protein expression level of **(a-b)** KCNJ10 in *Kcnj10*^+*/*+^(*n* = 2) and *Kcnj10*^*tm1a/tm1a*^ (*n* = 2); **(c-d)** KCNE1 in *Kcne1*^+*/*+^ (*n* = 2) and *Kcne1*^*tm1a/tm1a*^ (*n* = 2). All samples were run alongside GAPDH as a loading control **(a**,**c)**. The relative average intensity of protein bands was normalised to GAPDH and data are presented as the mean with individual datapoints overelaid **(b**,**d)**. Full size immuno blots are shown in supplementary figure 1; where technical replicates were available, the mean of the two measures was plotted in **b** and **d**. The expected molecular weights for each protein are as follows: KCNJ10: 41 kDa; KCNE1: 15 kDa; GAPDH: 36 kDa. Wildtype controls in this figure were 8 weeks old, *Kcnj10*^*tm1a/tm1a*^ mice were 8 weeks old and *Kcne1*^*tm1a/tm1a*^ mice were 6 weeks old. **(e)** Immunohistochemistry using DAB staining against COL4A3 in paraffin wax sections of the inner ear. A representative cross-sectional image of the stria vascularis showing COL4A3 staining in the strial capillary basement membranes of *Col4a3*^+*/*+^ (*n* = 3) and *Col4a3*^*tm1a/tm1a*^ mice (*n* = 2). Antigen sites appear brown, and nuclei are blue. Samples for immunohistochemistry were 4 weeks old.

To understand whether incomplete knockdown of transcript in the inner ear would also lead to reduced levels of protein expression, we performed Western blots on whole inner ear tissue from *Kcnj10*^*tm1a/ tm1a*^and *Kcne1*^*tm1a/tm1a*^ mice compared to wildtype controls (Fig. 10). For *Kcnj10*^*tm1a/tm1a*^ mice, we observe a reduction in the protein expression relative to GAPDH in the inner ear (Fig. 10a,b). However, for *Kcne1*^*tm1a/ tm1a*^ mice, results were variable but we observe no difference in the level of protein expression compared to wildtype values (Fig. 10c,d). For *Col4a3*^*tm1a/tm1a*^ mice, DAB staining was undertaken in paraffin sections of inner ear tissue as the antibody did not work in Western blotting. We observe no COL4A3 staining around the strial capillaries in *Col4a3*^*tm1a/ tm1a*^ mice, whilst clear labelling is visible around blood vessels in wildtype littermate controls (Fig. 10e), suggesting that COL4A3 protein levels are reduced in these mutants. Overall, this suggests that for KCNE1, wildtype levels of protein expression can be provided from a reduced level of mRNA, whilst the amount of mRNA is a rate-limiting factor for KCNJ10 and COL4A3 protein expression. In all three cases, hearing is normal in these mutants, suggesting that the level of gene and protein expression required to maintain normal hearing is gene specific.

### 3.6 Kcne1^tm1a/tm1a^ mice have a normal cardiac QT interval

**Fig. 11:**
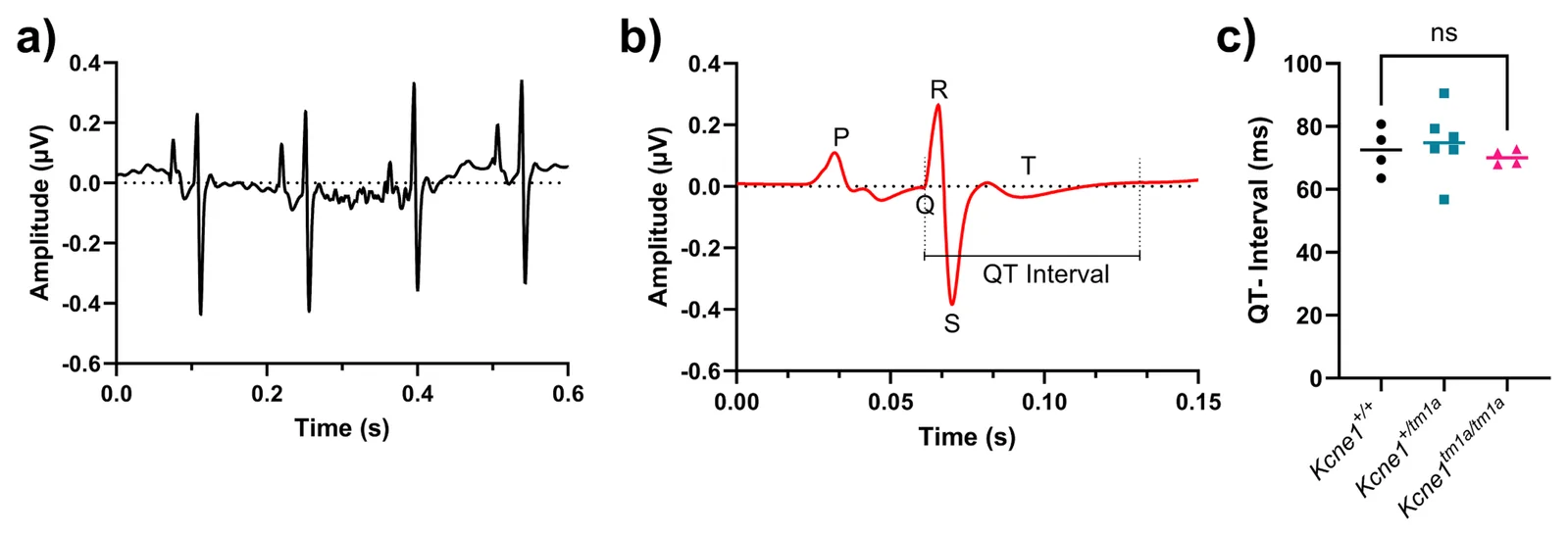
*Kcne1*^*tm1a/tm1a*^ mice have a normal QT interval at 4 weeks of age. **(a)** Representative trace from four consecutive ECG waveforms and **(b)** an averaged ECG waveform from a *Kcne1*^+*/*+^ mouse. **(c)** Analysis of the QT interval of 4 week old *Kcne1*^*tm1a/tm1a*^ mice (*n* = 4) compared to *Kcne1*^+*/*+^ (*n* = 4) and *Kcne1*^+*/tm1a*^ mice (*n* = 6). Data presented as individual data points with bar showing the mean. Comparisons were tested for significance using a One-way ANOVA.

Patients with JLNS have a prolonged cardiac QT interval measured from ECG recordings (Faridi et al., 2019). For this reason, *Kcne1*^*tm1a/tm1a*^ mice were tested for evidence of this phenotype (Fig. 11). ECG recordings from 4 week old *Kcne1*^*tm1a/tm1a*^ mice show that there is no significant difference in the duration of the QT interval compared to WT littermate controls (*p* = 0.70, One-way ANOVA) suggesting that *Kcne1*^*tm1a/tm1a*^ mice do not show evidence of this symptom of JLNS.

### 3.7 Col4a3^tm1a/tm1a^ mice show evidence of mild kidney impairment by 6 months of age

**Fig. 12:**
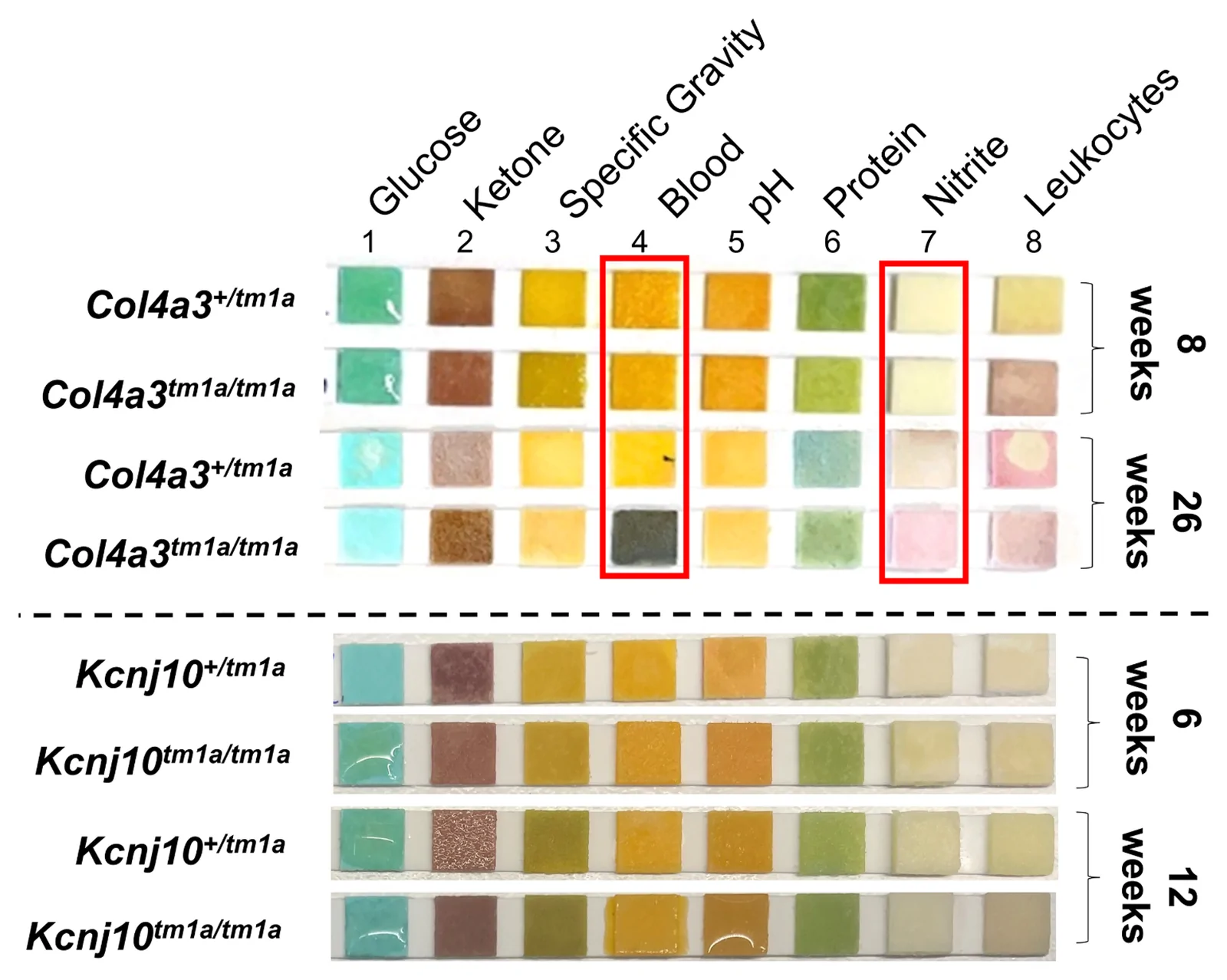
*Col4a3*^*tm1a/tm1a*^ mice have haemolysed blood in urine at 6 months of age. The results of urine testing *Col4a3*^*tm1a/tm1a*^ mice (*n* = 3; one male, two females) and heterozygous littermate controls (*n* = 4; two males, two females) at both 8 weeks and 6 months for signs of Alport kidney phenotype. Results from heterozygotes were within the normal colour range of the sticks. The results of urine testing in *Kcnj10*^*tm1a/tm1a*^ mice (*n* = 4, two males, two females) and littermate controls (*n* = 3, one male, two females) at both 6 and 12 weeks of age are also shown (lower panel).

A hallmark feature of Alport syndrome is kidney impairment which progresses from proteinuria in early stages to complete renal failure (Kleppel, Kashtan, et al., 1989; Mochizuki et al., 1994). *Col4a3*^*tm1a/tm1a*^ mice were tested for evidence of this phenotype using Siemens 8 G Multistix reagent strips. At 8-weeks of age both *Col4a3*^+*/tm1a*^ and *Col4a3*^*tm1a/tm1a*^ mice showed normal levels of all tested fractions of urine (Fig. 12). This was determined by a similar colour of test paper compared to littermate controls as well as compared to the colour chart supplied with the strips (see https://www.siemens-healthineers.com/en-uk/urinalysis-products/stix-family). However, at 6 months of age, *Col4a3*^*tm1a/tm1a*^ mice urine is positive for nitrite and there is evidence of a large amount of haemolysed blood in the urine (red box) indicative of early kidney failure. Both male and female mice were tested and both showed evidence of haemolysed blood at 6 months of age. We also tested *Kcnj10*^*tm1a/tm1a*^ mice for signs of abnormal urine components at 6 and 12 weeks of age because previous studies reported evidence of renal salt wasting in EAST patients (Bockenhauer et al., 2009). In both 6 week old and 12 week old mice we did not observe abnormalities in urine compared to littermate controls (Fig. 12).

## 4 Discussion

Here, we report the characterisation of three mouse lines which carry mutations in genes known to be associated with human deafness (*Kcnj10*^*tm1a*^, *Kcne1*^*tm1a*^ and *Col4a3*^*tm1a*^) to investigate whether they would develop an auditory phenotype similar to that of human patients with mutations in these genes, and to assess their potential for hearing loss reversal using treatments such as gene therapy. The three genes are expressed in different cell types of the stria vascularis. Surprisingly, all three mutants have normal ABR thresholds and wave 1 amplitude and latency up until 6 months of age. This finding contrasts with previous reports of hearing impairment where different mutations in these genes have been investigated.

In mice, deletion of exon 2 of *Kcne1* (*Kcne1*^*tm1Sfh*^ on either a 129S1/ Sv or a mixed 129S1/Sv;C57BL/6J genetic background) resulted in early postnatal collapse of the endolymphatic compartments of the inner ear with profound deafness, circling as well as head tossing behaviour by 4 weeks of age (Vetter et al., 1996; Wu et al., 2021). A spontaneous nonsense mutation *C* > *T* (p.Arg67*; *Kcne1*^*pkr*^) in mouse *Kcne1* was also reported and homozygous mutants were found to display a similar phenotype of deafness and vestibular abnormalities (Letts et al., 2000). Conversion of the *Kcne1*^*tm1a*^ allele (on a C57BL/6N genetic background with an unrepaired *Cdh23*^*ahl*^ allele) to the *Kcne1*^*tm1b*^ allele, by deletion of critical coding exon 2, leads to complete deafness at 14 weeks old as well as balance defects (Birling et al., 2021) (https://www.mousephenotype.org/data/genes/MGI:96673). We did not observe any obvious abnormal behaviour in *Kcne1*^*tm1a/tm1a*^ mice suggesting normal vestibular function, in addition to the normal auditory responses.

*Kcnj10*^*tm1Lst*^ homozygous mice on a mixed genetic background, where exon 2 was deleted and replaced with a neomycin resistance gene, had a reduced startle response (Rozengurt et al., 2003). In contrast to our finding of normal EP values from 8 week old *Kcnj10*^*tm1a/tm1a*^ mice, EP was abolished in 3 week old *Kcnj10*^*tm1Lst*^ homozygotes on a mixed 129/Sv;C57BL/6J genetic background (Marcus et al., 2002). *Kcnj10*^*tm1b*^ homozygotes on a C57BL/6N background die between embryonic day 18 and weaning (https://www.mousephenotype.org/data/genes/MGI:1194504), which is similar to reports of early pre-weaning death of *Kcnj10*^*tm1Lst*^ homozygotes (Bockenhauer et al., 2009). Our finding of normal auditory responses in *Kcnj10*^*tm1a*^ homozygotes together with normal viability suggests that the reduced level of mRNA and protein expression is enough to rescue the phenotype described in other, more severe, alleles.

Some Alport syndrome patients have normal hearing (Boeckhaus et al., 2020), demonstrating that normal hearing in *Col4a3*^*tm1a/tm1a*^ mice align with some patient reports. However, it is unclear whether these patients go on to develop hearing impairment with increased age because follow up studies are limited. Very mild hearing loss has also been observed in *Col4a3*^*tm1Dec/tm1Dec*^ mice, where a neomycin cassette was inserted into exon 5, on a 129/Sv genetic background between 6 and 8 weeks of age (Cosgrove et al., 1998) but not in mutant mice at 3 weeks of age (Dufek, Meehan, Delimont, Samuelson, et al., 2020). Some but not all individual *Col4a3*^*tm1Jhm*^ homozygotes on a 129/Sv background at 89–96 days old also showed raised ABR thresholds (Miner and Sanes, 1996). EPs recorded from *Col4a3*^*tm1Dec/tm1Dec*^ mice also show a small reduction (Smyth et al., 2024). Interestingly, the *Col4a3*^*tm1b/tm1b*^ mutant where exons 19 and 20 are deleted on a C57BL/6NTac background does not show evidence of hearing impairment at 14 weeks old (Birling et al. 2021; https://www.mousephenotype.org/data/genes/MGI:104688). The genetic background of the mouse may influence the development of an auditory phenotype, as has been described for other phenotypes of *Col4a3* mutants (Andrews et al., 2002; Irion et al., 2022) and may partially explain why *Col4a3*^*tm1a/tm1a*^ mice did not show evidence of hearing impairment.

The *Col4a3*^*tm1a*^ mutation that we studied was on a C57BL/6N genetic background which was repaired for the *Cdh23*^*ahl*^ allele, which might have led to normal hearing in our cohort. To investigate this further, we tested the hearing of *Col4a3*^*tm1a/tm1a*^ mice on the original C57BL/6N genetic background carrying the *Cdh23*^*ahl*^ allele, but these homozygotes also showed no sign of hearing impairment up to 6 months old.

A reduction in gene expression to around 25 % of wildtype values was observed in all three mutant mice homozygous for the tm1a allele. Despite this reduction, no hearing impairment was observed suggesting that 25 % of normal gene expression is sufficient to preserve auditory function. We measured expression levels in the whole inner ear, but we assume that every cell that would normally express each gene showed a similar reduced expression level. In gene therapy experiments, a reduced overall level of expression may result from normal expression levels in only targeted cells. For example, one study used a surgical canalostomy approach to deliver a high dose of *Kcne1* sequence to neonatal *Kcne1*^*tm1Sfh*^ homozygotes and 75.7 % of marginal cells and 36 % of vestibular dark cells were transfected, which was sufficient to prevent the development of hearing or balance impairment in these mice (Wu et al., 2021). Furthermore, in some cases the protein may be very stable (*e.g*. COL4A3) and a reduced amount of mRNA may produce enough protein to sustain function over the longer term. We found reduced COL4A3 and KCNJ10 protein levels in the mutants, but in the *Kcne1*^*tm1a*^ homozygotes the level of protein in whole inner ear tissue was similar to that in controls. Overall, these findings demonstrate that significantly less gene expression than is present in wildtype individuals can prevent the onset of hearing impairment.

At 8 weeks of age, abnormal arrangements of marginal cells in the stria vascularis were observed in the most apical regions examined (3 kHz) of all mutant mice as well as littermate controls, suggesting that this is a normal feature of strial morphology. Despite having normal hearing at this age, discrete gaps in the SV were observed along the entire length of the stria vascularis in *Kcnj10*^*tm1a/tm1a*^ and *Kcne1*^*tm1a/tm1a*^ mice. *Kcne1*^*tm1Sfh*^ homozygotes aged four weeks showed an abnormal arrangement of marginal cells that lacked a symmetrical hexagonal outline, but gaps similar to those we saw in *Kcne1*^*tm1a/tm1a*^ mice were not reported (Wu et al., 2021). It appears that this limited level of disorganisation of the marginal cell luminal surfaces is compatible with normal production of EP.

Another key feature of JLNS is a prolonged cardiac QT interval (Friedmann et al., 1966). *Kcne1* is expressed highly in mouse cardiac tissue (Drici et al., 1998; Felipe et al., 1994), and its current shows slow activation kinetics which are triggered by depolarisation (Barhanin et al., 1996). Therefore, *Kcne1*^*tm1a/tm1a*^ mice were tested for evidence of this phenotype. We did not observe a prolonged QT interval in mutant mice at 4 weeks of age. One study found that *Kcne1*^*tm1Sfh/tm1Sfh*^ mutants, where exon 2 was deleted, had a mildly prolonged QT interval during bradycardia (150 bpm), but not during faster heart rates (600 bpm) (Drici et al., 1998). This adaptability of the QT interval has also been reported in human patients with JLNS (Hirao et al., 1996; Neyroud et al., 1998), but not *in vitro* studies of isolated myocardium tissue (Charpentier et al., 1998). However, these authors noted that adaptation recorded from a restricted area of the myocardium was unlikely to correlate with the global activity of the ventricles *in vivo*. One suggestion for the adaptation observed *in vivo* has been that the fast-activating repolarising *K*^+^ current produced by *Kcnq1* may be sufficient to preserve the QT interval at higher heart rates (Warth and Barhanin, 2002). In our recordings, the average heartrate for *Kcne1*^*tm1a/tm1a*^ mice was 288 bpm, which may not have been slow enough to observe a prolonged QT interval in these mice. Differences in anaesthetic agent used such as sodium pentobarbital in Drici et al. or ketamine/xylazine, this study, may also produce different effects on the cardiovascular system and the duration of the QT interval recorded.

Type IV collagen is expressed in the renal basement membrane (Butkowski et al., 1989; Kleppel, Santi, et al., 1989). Alongside hearing impairment, haematuria and progressive renal failure are another hallmark feature of Alport syndrome (Gubler et al., 1981). Heterozygous carriers of *COL4A3* mutations in humans show an increased risk of kidney dysfunction including haematuria, and people with glycine missense mutations affecting the collagenous domain showed more severe phenotypes than people with null alleles (Solanki et al. 2023). However, heterozygous *COL4A3* mutations were not associated with hearing impairment (Solanki et al. 2023), suggesting that hearing loss is inherited in a recessive manner in these cases. In our study, no signs of kidney impairment were found in *Col4a3*^*tm1a/tm1a*^ mice at 8 weeks of age, but there was evidence of haematuria at 6 months of age in homozygotes while heterozygotes appeared normal. Renal impairment, due to thickening of the glomerular basement membrane and haematuria, can be observed as early as 4 weeks of age in *Col4a3*^*tm1Jhm*^ homozygous mice (Miner and Sanes, 1996) and death due to end stage renal disease seen by 14 weeks of age (Cosgrove et al., 1996). This contrasts significantly with the finding that *Col4a3*^*tm1a/tm1a*^ mice on a C57BL/6N genetic background can age healthily to 6 months of age. It is likely that residual protein expression and differences in genetic background underlie the phenotypic differences of *Col4a3*^*tm1a/tm1a*^ mice compared to knockouts.

The finding of signs of renal dysfunction in 6-month old *Col4a3*^*tm1a/ tm1a*^ homozygotes suggests that these mice might show delayed onset of other features of the phenotype including late-onset hearing loss, and this would be a useful area for future study. Furthermore, any of the mutants we analysed here could show increased susceptibility to environmental insults such as noise. Indeed, another *Col4a3* mouse mutant shows increased susceptibility to noise-induced damage (Meehan et al. 2016).

This report demonstrates that relatively low levels of expression of three known deafness genes (*Kcne1, Kcnj10* and *Col4a3*) expressed in different cell types of the stria vascularis may be sufficient to preserve hearing in patients with deafness associated with mutations in these genes. The finding that such limited amounts of expression can sustain normal auditory thresholds, together with our earlier finding that deafness due to another form of strial defect can be reversed (Martelletti et al. 2023), suggests that strial defects in general may be good targets for the development of treatments including gene therapy.

## Supplementary Material

Supplementary data Supplementary material associated with this article can be found, in the online version, at doi:10.1016/j.heares.2025.109356.

## Figures and Tables

**Table 1:** Primers used for short-range PCR genotyping of mouse lines.

Reaction name	Forward Primer	Reverse Primer	Size of band (bp)	Interpretation
**Kcnj10_wildtype**	CAT TCC TCC TTT CAG CAA CC	GCA GAG CCC TTG TTA AAT GC	239	Wildtype *Kcnj10* allele present
**Kcnj10_cassette**	CAT TCC TCC TTT CAG CAA CC	GAA CTT CGG AAT AGG AAC TTC G	144	*tm1a* allele present at *Kcnj10* locus
**Kcne1_wildtype**	AGG CAT CAG TTC TCT CGT CC	CAA CCC CTG TCA AAC ACT CG	193	Wildtype *Kcne1* allele present
**Kcne1_cassette**	AGG CAT CAG TTC TCT CGT CC	TCG TGG TAT CGT TAT GCG CC	169	*tm1a* allele present at *Kcne1* locus
**Col4a3_wildtype**	CCC TAC CTG CCT TCA CTG AT	TTT ATG GGC TTT CAG GTA GAA AC	264	Wildtype *Col4a3* allele present
**Col4a3_cassette**	CCC TAC CTG CCT TCA CTG AT	GAA CTT CGG AAT AGG AAC TTC G	142	*tm1a* allele present at *Co4a3* locus
**Neo**	CAA GAT GGA TTG CAC GCA GGT TCT C	GAC GAG ATC CTC GCC GTC GGG CAT GCG CGC C	546	Confirmation that *tm1a* allele is present

## Data Availability

Data will be made available on request.
