# Supplementary material for: Low level of expression of known deafness genes Kcne1, Kcnj10 or Col4a3 is sufficient to maintain hearing in mice
Source: Hear Res. Author manuscript; Available in PMC 2026 Sep 11. (PMC7619435; doi:10.1016/j.heares.2025.109356)
Supplement: Supplementary file 1 [file EMS213876-supplement-Supplementary_data.pdf]

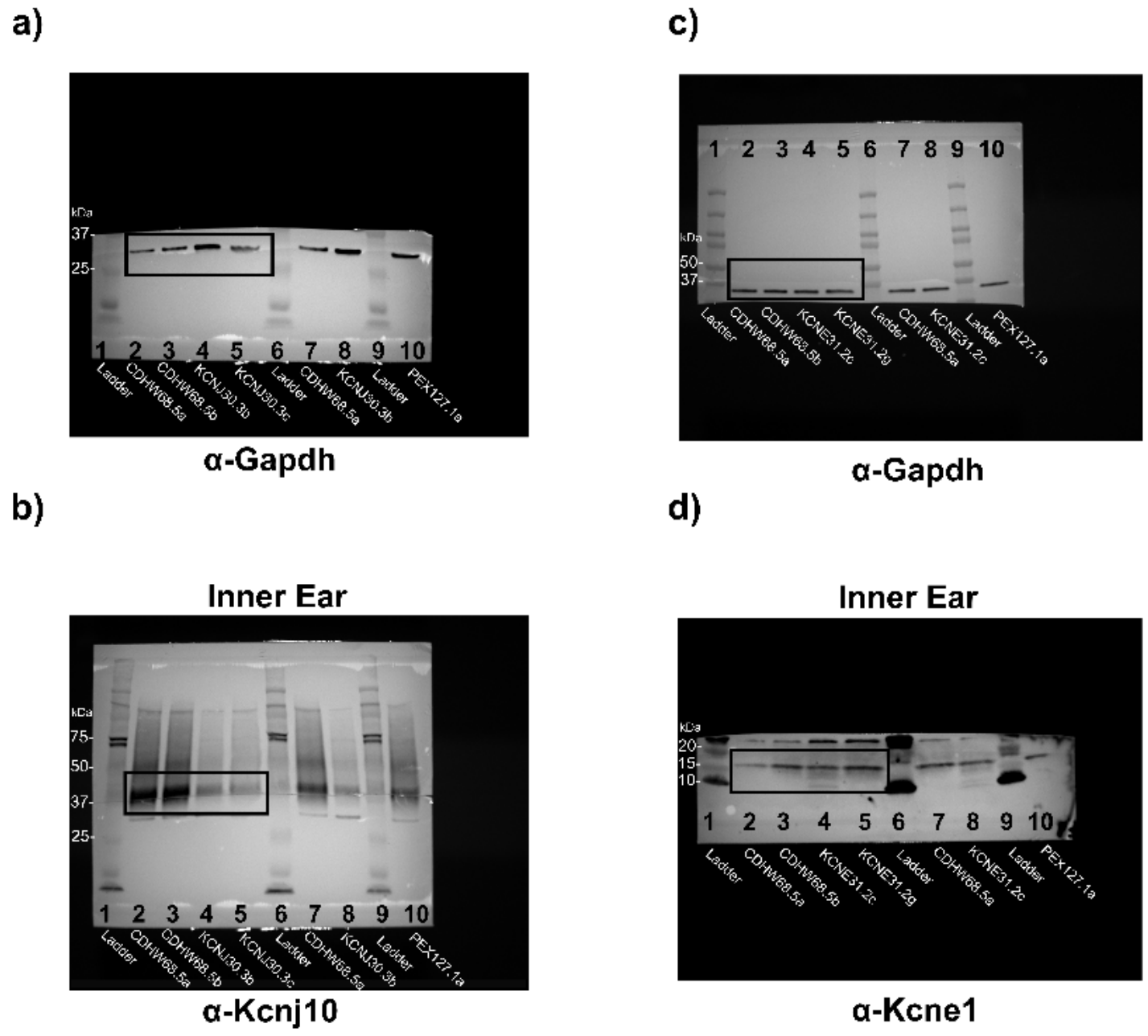

Supplementary Figure 1. Immuno blots used for quantification of KCNJ10 and KCNE1 protein in the inner ear. The full-size blots showing results of KCNJ10 and GAPDH loading control in the inner ear **(a-b)**. The full-size blots showing results of KCNE1 and GAPDH loading control in the inner ear **(c-d)**. Where available, technical replicates were averaged for analysis of protein quantification. The molecular weight of the ladder is shown to the left-hand side of each blot. The expected molecular weights for each protein are as follows: KCNJ10: 41 kDa; KCNE1: 15 kDa; GAPDH: 36 kDa. Wildtype controls in this figure (CDHW68.5a and b) were 8 weeks old, *Kcnj10*$^{tm1a/tm1a}$ mice (KCNJ30.3b and c) were 8 weeks old and *Kcne1*$^{tm1a/tm1a}$ mice (KCNE31.2c and g) were 6 weeks old. One additional wildtype control sample (PEX127.1a) was added to each blot but was not used for quantification purposes. The black box represents the images shown in Figure 10.
